# Crowdsourcing punishment: Individuals reference group preferences to inform their own punitive decisions

**DOI:** 10.1038/s41598-019-48050-2

**Published:** 2019-08-12

**Authors:** Jae-Young Son, Apoorva Bhandari, Oriel FeldmanHall

**Affiliations:** 1 Department of Cognitive, Linguistic, and Psychological Sciences, Brown University, Providence, RI 02912 USA; 2 Carney Institute for Brain Science, Brown University, Providence, RI 02912 USA

**Keywords:** Psychology, Human behaviour

## Abstract

Justice systems delegate punishment decisions to groups in the belief that the aggregation of individuals’ preferences facilitates judiciousness. However, group dynamics may also lead individuals to relinquish moral responsibility by conforming to the majority’s preference for punishment. Across five experiments (N = 399), we find Victims and Jurors tasked with restoring justice become increasingly punitive (by as much as 40%) as groups express a desire to punish, with every additional punisher augmenting an individual’s punishment rates. This influence is so potent that knowing about a past group’s preference continues swaying decisions even when they cannot affect present outcomes. Using computational models of decision-making, we test long-standing theories of how groups influence choice. We find groups induce conformity by making individuals less cautious and more impulsive, and by amplifying the value of punishment. However, compared to Victims, Jurors are more sensitive to moral violation severity and less readily swayed by the group. Conformity to a group’s punitive preference also extends to weightier moral violations such as assault and theft. Our results demonstrate that groups can powerfully shift an individual’s punitive preference across a variety of contexts, while additionally revealing the cognitive mechanisms by which social influence alters moral values.

## Introduction

In the classic film *Twelve Angry Men*, a jury must decide whether to convict a defendant accused of murder, thereby condemning him to death. In a memorable scene, Juror #8 hesitantly looks around the table as the initial vote is being taken by a show of hands, then conforms to the majority by voting ‘guilty.’ Why did the juror look to his peers when considering his vote? As in the film, it could be that we turn to others for guidance about how to enact justice in real-world contexts. Decades of conformity experiments demonstrate that individuals’ behaviors, preferences, and beliefs can be swayed by their peers in domains as wide-ranging as visual perception, inferences about socially normative behaviors, intensity of pain experience, preferences for food and music, and the attractiveness of faces^1–7^. Furthermore, recent evidence from both lab and field studies suggests that people behave more prosocially after observing others engaging in behaviors such as cooperation and generosity^8–10^.

However, since moral values appear to be deeply-held, stable across time, and resistant to change^11–13^, a parallel literature on moral cognition makes a different prediction: insofar as punishment is a method of restoring justice following a moral violation, punitive preferences should be less susceptible to group influence. For example, people form more extreme judgments of an action’s rightness or wrongness when primed to perceive the action as having a moral dimension^14^, which results in a stronger entrenchment of moral attitudes^15^. In field studies, moralized attitudes lead to decreased support for political compromise^16^ and contribute to the intractability of conflicts in which sacred values are at stake^17^. Indeed, it appears that moral attitudes may be an immutable feature of one’s individual identity^18^, and are therefore unwavering across social contexts.

Given these divergent predictions, an important goal is to test the susceptibility of moral attitudes—in this case, punishment preferences—to group influence, and to understand the social and cognitive factors that might lead an individual to conform to a group’s desire to punish. Understanding the malleability of punitive preferences is especially important given that punishment of norm violators is a pervasive part of human social life^19–21^. Moreover, punishment decisions are often made within group contexts^22^, and are decided both by those who have been directly affected by a crime (i.e., victims), and by those who are impartial third parties (i.e., jurors).

Victims, for example, are often given the ability to recommend (or even determine) punishment across a wide variety of social settings: the submission of impact statements as evidence in a courtroom^23^, deployment of informal or quasi-judicial practices in institutional contexts such as universities based on the responses of many victims^24^, tribal and religious councils^25^, war tribunals where punishment recommendations are made collectively by victims^26^, and even instances when people employ vigilante justice^27^. These real-world decisions about justice are sometimes made within contexts where groups have the ability to sway a victim’s desire for punishing a perpetrator, as is commonly observed when crowd dynamics lead to escalated violence during riots^28,29^.

A juror—who is tasked with making punishment decisions on behalf of victims—typically also makes a punishment recommendation within the context of a group, which introduces the possibility that an individual juror can be swayed by others’ preferences^30^. One large-scale field study surveyed nearly 3,500 real jurors who had actually decided upon felony cases and found that over one-third of them would have reversed their jury’s decision if they had been given sole control over the trial’s outcome^31^. Though observational studies such as these are limited in their ability to establish causal mechanisms, this finding provides preliminary evidence that a group’s preference can have a powerful influence on one’s willingness to punish. This is an especially important phenomenon to understand if we consider that wrongful punishment has catastrophic and irreversible consequences^32^. As a vivid illustration of the potential consequences of conformity in punishment decisions, we return to the fictional example from *Twelve Angry Men*, in which all but one juror initially voted to convict. Were it not for this standalone juror who refused to be swayed by the majority, the defendant—proven innocent at the end of the film—would have been wrongfully sentenced to death.

To investigate the psychological factors and cognitive mechanisms influencing one’s willingness to endorse punishment as a means of restoring justice, we leverage an interdisciplinary approach, drawing from social psychology, behavioral economics, and computational modeling. We first tested whether individuals’ punitive preferences are malleable when they are a victim of a moral violation (Experiments 1–2). Based on classic conformity research showing that individuals are sensitive to the proportion of people within a group who endorse an option in a non-moral context^1^, we examined whether victims’ punishment rates scale with the proportion of punitive preferences expressed within a group (Experiment 1). We further investigated the strength of social influence by examining whether victims’ punishment behaviors continue to be affected by a *past* group’s punitive preferences, even when that past group’s preference is objectively irrelevant to the current decision (Experiment 2). Given existing work illustrating that people punish norm violators both as a second-party victim or third-party juror^19,20,33^, we then tested whether an individual’s susceptibility to group influence hinges on whether they are a wronged victim or an unaffected juror (Experiments 3–4). In addition to examining behavior, we employed a computational model of decision-making (the Drift Diffusion Model) to reveal how a group’s preferences influence the cognitive processes underlying punishment decisions when tasked with restoring justice. Finally, we assessed whether conformity to a group’s punitive preferences generalizes to a variety of other real-world moral transgressions (Experiment 5).

## Results

### Experiment 1: Susceptibility to a group’s punishment preferences as Last Decider

**Figure 1:**
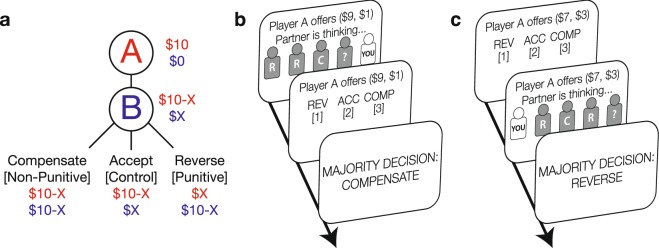
Task and trial structure for Experiment 1 (Last Decider) and 2 (First Decider). (**a**) In the modified Justice Game, Player A is endowed with $10 at the beginning of each trial. Player A offers some portion of that money with Player B, who can choose to Accept, Compensate, or Reverse the offer. In our task, Player As always made unfair offers. Because Player Bs could maximize their payout by choosing either Compensate or Reverse, decisions to Reverse can be interpreted as cost-free punishment for Player As. (**b**) Subjects in the Last Decider experiment observed their group’s punishment preferences prior to indicating their own punishment preference. (**c**) Subjects in the First Decider experiment indicated their punishment preference prior to observing their group’s preferences. Although group members are represented by icons in this figure, subjects were shown photographs in the actual experiment.

We first examined whether decisions to restore justice are malleable, and if so, whether these moral preferences are sensitive to the proportion of other individuals endorsing a punitive response in the wake of a fairness violation. Subjects completed a variant of the Justice Game, an economic task specifically designed to measure punitive and non-punitive responses to fairness violations^33^. In this task, Player A is endowed with $10 on each trial and can choose how much to split with the subject, who always takes the role of Player B (Fig. 1a). Player A’s splits ranged from mildly unfair (a 6/4 split in which Player A keeps $6 and offers $4) to highly unfair (9/1 split) in $1 increments. After receiving an unfair offer, Player B then decided how to restore justice by choosing to: (i) Accept Player A’s split as-is; (ii) Compensate themselves in a cost-free manner by increasing their own payout to match Player A’s payout, without punishing Player A; or (iii) Reverse Player A’s split, a highly retributive option that maximizes Player B’s own payout and minimizes Player A’s payout (Fig. 1a).

Subjects first completed a Solo Phase of this task, allowing us to measure subjects’ punishment preferences in the absence of any social influence. Accounting for the possibility that subjects might infer strategic motives behind Player A offers, payout at the end of the study was implemented probabilistically such that Player A’s original offer was enacted half of the time and Player B’s decision was enacted half of the time (see Supplementary Methods). Indeed, subjects Reversed at greater rates as offers became increasingly unequal, indicating that they perceived small offers as unfair and punished the perpetrator accordingly. Subjects played with new Player As on each trial.

Subjects then completed a Group Phase of the task, in which they were told that they were sharing the role of Player B alongside four other subjects, and that their responses would collectively determine the payouts of all the players (Fig. 1b). Specifically, subjects were informed that the payout redistribution would be determined by the majority choice of the five Player Bs, that Player A’s payout would be affected by Player Bs’ collective decision just as it had in the Solo Phase, and that each Player B would receive the full payout. As in the Solo Phase, each of the players sharing the role of Player B could choose to Accept, Compensate, or Reverse Player A’s split, and subjects observed the other Player Bs’ choices in sequence, as if they were made in real time (Fig. 1b). Subjects always responded last, following the format of the classic Asch conformity paradigm^1^. Subjects played with new Player As and Bs on each trial. To test the possibility that endorsement rates of the punitive Reverse option would increase as the number of punishers within the group increased, we parametrically and deterministically varied the proportion of players who selected the punitive option from 0% to 100%.

**Figure 2:**
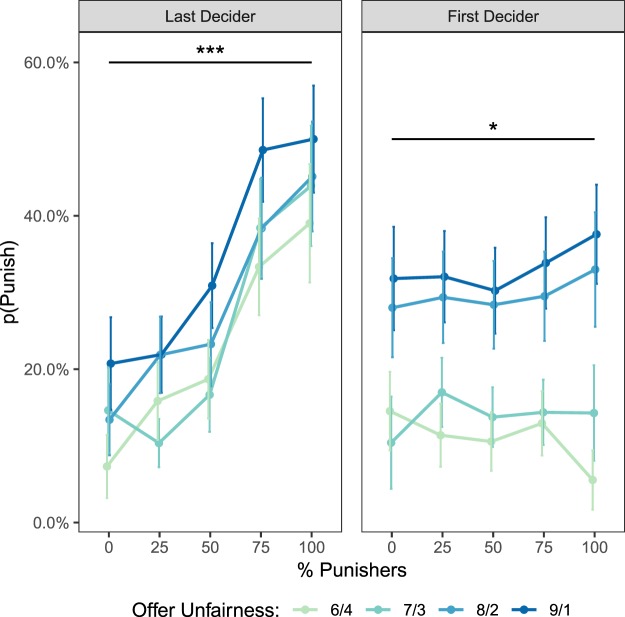
Results for Experiments 1 (Last Decider) and 2 (First Decider). For both experiments, preference for punishment increases with the proportion of punishers. This effect remains significant, albeit is reduced, when subjects are observing the moral preferences of past groups who cannot affect the current choice (First Decider). Error bars reflect ± 1 SEM.

To examine whether a group’s punitive preferences alter an individual’s willingness to punish, we conducted a mixed-effects logistic regression analysis. We modeled the probability of punishing as an additive combination of the proportion of punishers in the group (centered around 50% punishers), the unfairness of Player A’s offer (centered around medium unfairness), and each subject’s baseline preference for punishment (i.e., the proportion of Solo Phase trials in which a subject chose to Reverse instead of Compensate, matched for offer unfairness and centered around 50% punishment). Results reveal that individuals significantly increase punishment as a greater proportion of the group expresses a punitive preference, even after accounting for offer unfairness and baseline punitive preferences (Table 1; Fig. 2). This effect was so strong that the group was ultimately able to shift individuals’ predicted punishment rates as much as 30 percentage points.

**Table 1:** Experiment 1: Last Decider.

*Punishment*_*i*,*t*_ = *β*_0_ + *β*_1_ *Proportion of Punishers*_*i*,*t*_ + *β*_2_ *Offer Unfairness*_*i*,*t*_ + *β*_3_ *Baseline Punishment*_*i*,*t*_			
Term	Estimate (*SE*)	*z*	*p*
Intercept	−2.23 (0.46)	−4.82	<0.001***
Proportion of Punishers	1.06 (0.26)	4.04	<0.001***
Offer Unfairness	0.12 (0.03)	4.20	<0.001***
Baseline Punishment	0.82 (0.37)	2.20	0.028*

Note: Terms are indexed by subject (*i*) and trial (*t*). For each subject, the model includes a random intercept, a random slope for the proportion of punishers, and a random slope for baseline punishment. A fully maximal model including a random slope for offer unfairness led to model convergence failure, and we therefore exclude this term^50^.

*p < 0.05, **p < 0.01, ***p < 0.001.

### Experiment 2: Influence of past groups’ punishment preferences as First Decider

The findings from Experiment 1 are the first that we are aware of illustrating that individuals’ moral attitudes about punishment vary with the intensity of a group’s preference. To examine the robustness of this conformity effect and the potency of social influence, we conducted a second experiment examining the possibility that individuals might even conform to *previous* groups endorsing punishment. Specifically, we tested two competing hypotheses: whether the effect of social influence is abolished as soon as a past group’s preferences are no longer relevant to the current decision context^1,34^, or whether knowing that a past group sanctioned punishment would also affect how readily an individual chooses to punish in the present moment. Evidence for the second hypothesis would suggest that decisions to punish are highly malleable. To probe whether subjects’ punitive behaviors are susceptible to influence even from past groups’ preferences, subjects in Experiment 2 were first in their group to decide during the Group Phase (i.e. playing as the “First Decider”, in contrast to Experiment 1, in which subjects played as the “Last Decider”).

The methodology of Experiment 2 was identical to that of Experiment 1, with one key difference: the sequence of Player Bs in the Group Phase was fixed so that subjects were always the first person in the group to make their decision (Fig. 1c). When deciding first, subjects did not have access to their present group’s punitive preferences. Therefore, on any given trial, the only two pieces of information that could affect a subject’s decision were the unfairness of Player A’s offer and the preferences of past groups. Given that behavioral economic experiments examining fairness norms have found that people are sensitive to the magnitude of fairness violations, including in the Justice Game^33^, we defined the proportion of punishers in a way that would allow us to match fairness violations across trials. We employed a simple analysis approach such that on each trial, we recorded Player A’s fairness violation (from mildly unfair splits of $6/$4 to highly unfair splits of $9/$1), searched backwards until we found the most recent past trial in which the severity of the fairness violation matched that of the present trial, and then used the proportion of punishers observed on that past trial to predict decisions to punish on the current trial. Alternative models with different operationalization of trial history are discussed in the Supplementary Results; we report the best-fitting model here.

This task design also allows us to address two potential concerns about the validity of our paradigm: first, that explicit pressure to conform in Experiment 1 encouraged subjects to behave in ways they believed would please the experimenter, and second, that the observed conformity effect is only observable under conditions where people believe that their individual decision has little influence on the outcome of the group’s final decision. Therefore, in addition to testing whether punishment decisions remain susceptible to past groups’ influence, the task design of Experiment 2 also allowed us to determine whether the conformity effect replicates in a decision context where explicit pressure to conform is reduced and where subjects explicitly know their choice can affect the final outcome.

As in Experiment 1, results indicate that increasing the proportion of punishers within a group significantly enhances punishment endorsement rates, even when accounting for offer unfairness and baseline punitive preferences (Table 2; Fig. 2). Although the strength of the conformity effect was attenuated relative to when individuals were last to decide, our results illustrate that individuals still conform when their decisions are consequential and when they are relatively free from experimenter demand effects. An ancillary analysis also demonstrates our results are not significantly modulated by the time elapsed since the key trial used to define the proportion of punishers (Supplementary Results).

**Table 2:** Experiment 2: First Decider.

*Punishment*_*i*,*t*_ = *β*_0_ + *β*_1_ *Proportion of Punishers*_*i*,*t*_ + *β*_2_ *Offer Unfairness*_*i*,*t*_ + *β*_3_ *Baseline Punishment*_*i*,*t*_			
Term	Estimate (*SE*)	*z*	*p*
Intercept	−2.83 (0.64)	−4.40	<0.001***
Proportion of Punishers	0.28 (0.13)	2.24	0.025*
Offer Unfairness	0.47 (0.04)	11.77	<0.001***
Baseline Punishment	1.54 (0.57)	2.70	0.007**

Note: Terms are indexed by subject (*i*) and trial (*t*). The regression model specification is identical to Experiment 1: for each subject, the model includes a random intercept, a random slope for the proportion of punishers, and a random slope for baseline punishment.

*p < 0.05, **p < 0.01, ***p < 0.001.

### Experiment 3: Drift diffusion model of conformity as a Victim

Experiments 1–2 reveal just how powerfully social influence can alter individuals’ endorsement of punishment as a means of restoring justice. However, it remains an open question how social influence operates on the cognitive mechanisms underlying decisions to punish norm violators, thereby producing conformist behaviors. To explore this question, we performed a third experiment leveraging the Drift Diffusion Model (DDM), a computational model of decision-making. Though the DDM is typically used in perceptual decision-making contexts, it is well-suited for examining the effects of social influence, and has recently gained some traction within the social domain, successfully explaining how social groups bias perceptual judgments and even how altruistic decisions unfold^35,36^.

In the context of our task, DDM uses choices and reaction time distributions to characterize how people integrate evidence about the value of punishment (such as the group’s punitive preference) relative to compensation^37^. This allows us to decompose the decision-making process into psychologically-meaningful parameters. First, bias (*z*) quantifies the extent to which people lean towards one of the justice restoration options prior to observing any evidence. Second, decision threshold (*a*) indexes the amount of evidence subjects need in order to make a choice, thereby capturing how cautiously individuals make choices in the presence (and absence) of group influence. Third, drift rate (*v*) indexes the strength of evidence favoring either punishment or compensation obtained from observing the group’s preference, and therefore how much an individual weighs the value of punishment relative to the value of compensation^36,38^. Importantly, the strength of evidence in our task is not dependent on the dynamics of the stimulus (such as movement coherence in random dot motion tasks), but instead reflects the psychological process through which a group’s punitive preferences are dynamically integrated into an individual’s valuation of punishment.

Therefore, DDM enables us to rigorously test two longstanding hypotheses from social psychological theory about how group influence acts on the cognitive mechanisms governing decision-making. First, we can test whether the presence of a group majority lowers the stakes of an individual’s decision (as the subject is now merely one vote out of many), thereby reducing the total amount of evidence required to commit to a choice and leading the individual to relinquish moral responsibility. Second, we can test whether the proportion of people within a group endorsing a punitive option increases the strength of evidence that punishment is a valuable method for restoring justice.

**Figure 3:**
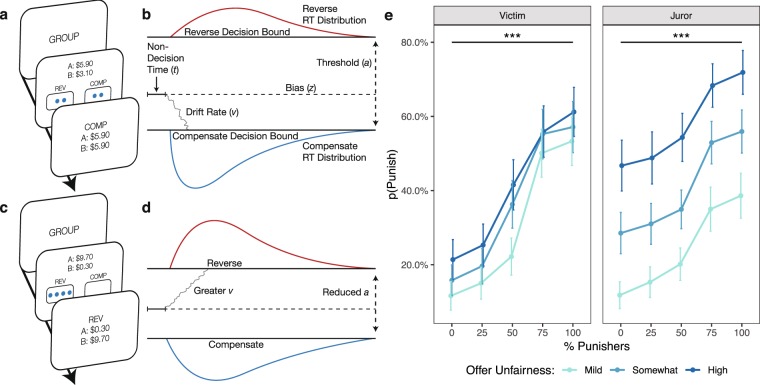
Schematics and behavioral results of Experiments 3 and 4 (Victim and Juror). (**a**) As in the Last Decider study, subjects in Experiments 3 and 4 observed their group’s punishment preferences prior to indicating their own punishment preference. In this example, the group is evenly split between punishers and compensators. (**b**) According to the Drift Diffusion Model (DDM), people make decisions by noisily accumulating evidence in favor of each of the options until a decision bound is reached. In this cartoon schematic, subjects are biased such that they initially prefer compensation over punishment, indicated by the starting point *z*; they require some threshold of evidence in order to commit to a choice, indicated by *a*; and they accumulate evidence in favor of compensation, indicated by *v*. Together, these parameters dictate the shapes of reaction time distributions for punishment and compensation. (**c**) In this example trial, the group unanimously prefers to punish the unfair offer. (**d**) We illustrate using a cartoon schematic how conformity to the group underlie shifts in the *v* and *a* parameters, thereby changing choice and reaction time distributions. (**e**) When deciding as either a Victim or Juror, preference for punishment increases with the proportion of punishers, replicating Experiment 1 (Last Decider). Error bars reflect ± 1 SEM.

Given that the greatest conformity effects occur when subjects are the last to decide within the group, Experiment 3 followed the same task structure as Experiment 1 (Last Decider), with a few key modifications to make the task suitable for DDM. First, because the best-established DDMs only account for binary choices, subjects were only presented with the Compensate and Reverse options on each trial. Second, subjects sometimes made decisions in the absence of information about others’ preferences during the Group Phase of the task (hereafter referred as the Alone condition), which allows us to avoid ordering confounds from the Solo Phase that might contaminate DDM parameters (e.g. from unrelated motor or task learning). Third, offers from Player As were placed within discrete monetary bins and jittered to offset task habituation from repeatedly seeing only four distinct offer types (as in Experiments 1–2), which was especially important given the large number of trials needed to estimate DDM parameters. Therefore, offers from Player As were binned into three levels of unfairness, all drawn from a uniform distribution in increments of 10¢: Mildly Unfair offers between $3.70 and $4.90, Somewhat Unfair offers between $1.90 and $3.10, and Highly Unfair offers between $0.10 and $1.30. Finally, because DDM requires reaction time (RT) distributions to capture the decision process in its entirety^37^, subjects were simultaneously presented with Player A’s offer and four randomly-sampled responses from other Player Bs (Fig. 3a,c; this is in contrast to Experiments 1–2, in which subjects observed Player A offers and Player B choices sequentially). Subjects were free to make a response at their own pace, but were encouraged to make decisions as quickly as possible.

Replicating the behavioral findings from Experiment 1, results indicate that the group’s punitive preferences significantly affects endorsement of punishment (Table 3; Fig. 3e), shifting individuals’ punitive choices by as much as 40 percentage points. We then used the HDDM software package to perform hierarchical Bayesian estimation of DDM parameters^39^. The drift rate (*v*), threshold (*a*), and bias (*z*) parameters were estimated at the group level using a hierarchical Bayesian procedure. This allows individuals’ contributions to the group parameters to be weighted according to their diagnostic value, maximizes power by capitalizing on statistical similarities between subjects, and addresses collinearity between variables by incorporating greater uncertainty in the posteriors of parameter estimates. To additionally account for within-subject variability, the proportion of punishers was regressed onto the DDM parameters of interest. Three regressions were performed, one for each bin of offer unfairness. Decisions to punish were mapped to the upper boundary, and decisions to compensate were mapped to the lower boundary (Fig. 3b). Separate parameters were fit for each bin of offer unfairness. Drift rate and threshold were allowed to vary by the proportion of punishers in the group, whereas a single bias parameter was estimated for each type of unfairness. This model specification reflects that people likely have preexisting biases about how much punishment is warranted depending on the degree of fairness violations, while making no claims that preexisting bias varies according to group dynamics. Alternatively-specified models are tested and discussed in the Supplementary Information.

**Table 3:** Experiment 3: Punishing as Victim.

*Punishment*_*i*,*t*_ = *β*_0_ + *β*_1_ *Proportion of Punishers*_*i*,*t*_ + *β*_2_ *Offer Unfairness*_*i*,*t*_ + *β*_3_ *Baseline Punishment*_*i*,*t*_			
Term	Estimate (*SE*)	*z*	*p*
Intercept	−1.18 (0.17)	−7.11	<0.001***
Proportion of Punishers	1.50 (0.27)	5.63	<0.001***
Offer Unfairness	−0.09 (0.02)	−4.27	<0.001***
Baseline Punishment	6.13 (0.42)	14.74	<0.001***

Note: Terms are indexed by subject (*i*) and trial (*t*). The regression model specification is identical to Experiments 1–2: for each subject, the model includes a random intercept, a random slope for the proportion of punishers, and a random slope for baseline punishment.

*p < 0.05, **p < 0.01, ***p < 0.001.

Statistical significance in the context of Bayesian estimation is determined by the proportion of values in a posterior distribution that fall above or below a point value, such as zero (i.e., testing whether a regression coefficient is different from zero), or the mean of another posterior distribution. Bayesian hypothesis testing is therefore conceptually akin to performing a frequentist t-test^40^. We define significance as 95% of posterior values falling above (or, depending on the analysis, below) the specified point value. To avoid confusion, we report 1–*p*, as this statistic more closely resembles p-values from frequentist significance testing.

First, given past findings from the Justice Game that Victims typically prefer to compensate in the wake of a fairness violation^33^, as well as our behavioral data from Experiments 1–2, we predicted that Victims would exhibit an overall bias in favor of choosing the compensatory option over the punitive one. This would be reflected by the bias (*z*) parameter being closer to the decision boundary for compensating (see Fig. 3b for a schematic). Values greater than 0.5 indicate an initial preference for punishment, and values less than 0.5 indicate an initial preference for compensation. Indeed, we found that Victims exhibit a preference for compensation over punishment, reflected by 95% of posterior *z* estimates falling under the point value 0.5 (average *z* for Mildly Unfair = 0.42, Somewhat Unfair = 0.43, Highly Unfair = 0.44, all posterior *Ps* < 0.001). This indicates that highly-punitive groups were able to influence Victims’ behaviors despite individuals having a predisposition not to punish. This preference for compensation did not significantly vary as a function of offer unfairness (all posterior *Ps* > 0.10).

**Figure 4:**
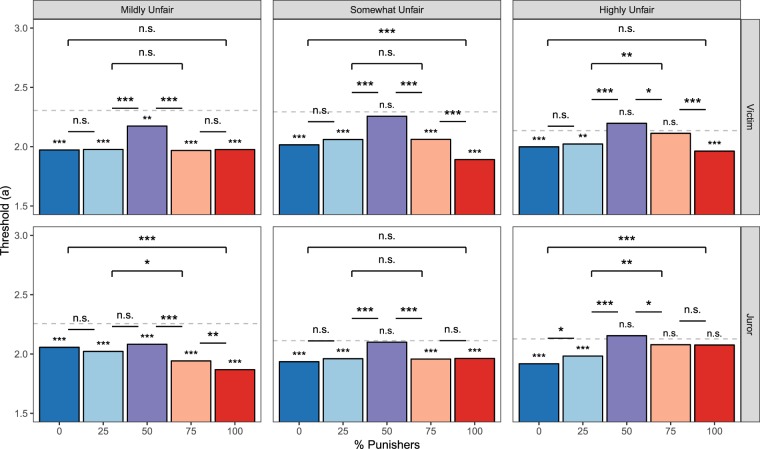
Mean threshold parameter estimates for Victim and Juror DDM experiments. Significance asterisks above/below each bar represent differences from the Alone condition (which was used as the reference category and is represented by a grey dashed line in each panel), and lines indicate pairwise differences. Threshold is estimated to be higher for decision contexts in which subjects determine the final outcome (i.e., choosing alone and with evenly-split groups).

Second, we predicted that individuals would be less cautious within groups where a majority of group members endorse punishment or compensation, as the individual would only be one vote of many. This would be reflected in a decrease in the distance *a* between decision thresholds (Fig. 3d). Consistent with our hypothesis, results reveal that Victims have a lowered average decision threshold when making decisions within a group majority, relative to a group that is evenly split between punishers and compensators (all posterior *Ps* < 0.001; Fig. 4). We additionally find that individuals are generally less cautious and more impulsive when choosing within a group majority, relative to when they are choosing alone (Fig. 4). Average *a* did not differ as a function of offer fairness (all posterior *Ps* > 0.40).

**Figure 5:**
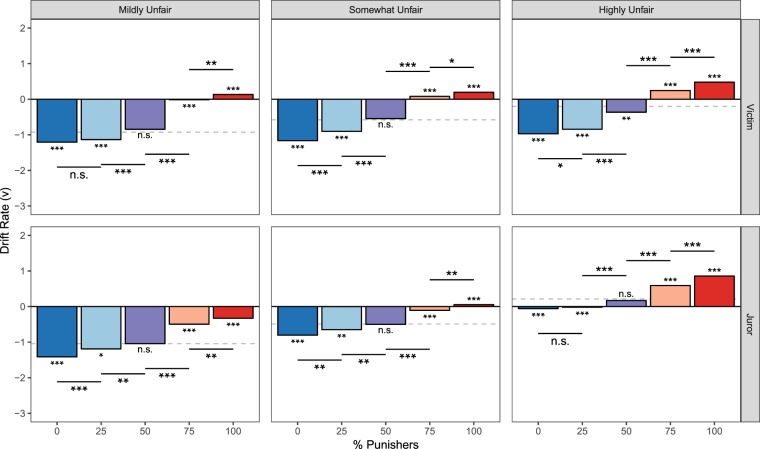
Mean drift rate parameter estimates for Victim and Juror DDM experiments. Significance asterisks above/below each bar represent differences from the Alone condition (which was used as the reference category and is represented by a grey dashed line in each panel), and significance bars indicate pairwise differences. Drift rates grow increasingly positive with the proportion of punishers, indicating that people accumulate evidence favoring punishment from groups.

Third, because greater group endorsement of the punitive option provides individuals with stronger evidence favoring punishment, we predicted that drift rate (*v*) would increase as the proportion of punishers in the group increased. The magnitude of *v* indicates the strength of evidence favoring each option, while the sign of *v* indicates whether the evidence favors punishment (positive) or compensation (negative; see Fig. 3b,d for a schematic). Consistent with subjects’ average preference for compensation, drift rates are largely negative. However, *v* increases with the proportion of punishers, demonstrating that Victims used the proportion of punishers as accumulating evidence of punishment’s value (Fig. 5). Average *v* did not differ as a function of offer fairness (all posterior *Ps* > 0.05).

### Experiment 4: Drift diffusion model of conformity as a Juror

Experiment 3 replicates our finding that Victims’ punishment decisions are susceptible to the preferences of a group. In addition, these findings demonstrate that groups act upon the decision-making process by making individuals less cautious about their choices, and by increasing how much individuals value punishment as a method of restoring justice. However, a potential alternative interpretation of these results is that individuals are acting upon an existing desire to punish and are thus not conforming to a group’s punitive preferences. That is, Victims may be reluctant to punish norm violators because they believe that retribution is perceived as being socially undesirable; observing others being punitive would then enable them to act upon their latent desire to punish. To distinguish between these alternative interpretations—and to examine whether people also conform to a group’s punitive preferences when tasked with making third-party punishment decisions^19,20,30,31^—we conducted a fourth experiment that was identical to Experiment 3 with the major exception that subjects made punishment decisions as Jurors on behalf of victims.

Behavioral results indicate that the group’s punitive preferences significantly modulates punishment (Table 4), shifting predicted punishment by nearly 30 percentage points. A formal comparison between Experiment 3 (Victim) and Experiment 4 (Juror) additionally finds that the conformity effect is significantly weakened for Jurors relative to Victims (β = 0.50, *SE* = 0.13, *z* = −2.58, *p* = 0.010; estimate and SE are in units of odds ratios).

**Table 4:** Experiment 4: Punishing as Juror.

*Punishment*_*i*,*t*_ = *β*_0_ + *β*_1_ *Proportion of Punishers*_*i*,*t*_ + *β*_2_ *Offer Unfairness*_*i*,*t*_ + *β*_3_ *Baseline Punishment*_*i*,*t*_			
Term	Estimate (*SE*)	*z*	*p*
Intercept	−0.83 (0.12)	−7.07	<0.001***
Proportion of Punishers	0.69 (0.16)	4.43	<0.001***
Offer Unfairness	0.08 (0.02)	4.15	<0.001***
Baseline Punishment	6.10 (0.47)	13.09	<0.001***

Note: Terms are indexed by subject (*i*) and trial (*t*). The regression model specification is identical to Experiments 1–3: for each subject, the model includes a random intercept, a random slope for the proportion of punishers, and a random slope for baseline punishment.

*p < 0.05, **p < 0.01, ***p < 0.001.

In contrast to Victims (who were consistently biased in favor of compensation), DDM results reveal that Jurors were only biased in favor of compensation when fairness violations were Mildly Unfair (average *z* = 0.45, posterior *P* < 0.001). When offers were instead Somewhat or Highly Unfair, *z* was not statistically different from the neutral starting point 0.5 (average *z* for Somewhat Unfair = 0.48, Highly Unfair = 0.48, both posterior *Ps* > 0.05). This reveals that before observing the group’s preferences, Jurors were more neutral than Victims (i.e., not biased towards either compensation or punishment).

Replicating the pattern observed for Victims, Jurors exhibited a lowered decision threshold when choosing in a group majority than when choosing in an evenly-split group (all posterior *Ps* < 0.001), and generally when choosing in a group majority relative to choosing alone (with a noticeable exception for when a majority of the group chose to punish highly unfair offers; Fig. 4). Although the pattern of threshold estimates between Victims and Jurors appear to be qualitatively different upon visual inspection (Fig. 4), direct statistical comparisons of the *a* parameter reveals no significant difference between Victims’ and Jurors’ decision criterions when comparing average *a* for each bin of offer unfairness (all posterior *Ps* > 0.20). Average *a* did not differ as a function of offer fairness for Jurors (all posterior *Ps* > 0.30), as was the case for Victims.

**Figure 6:**
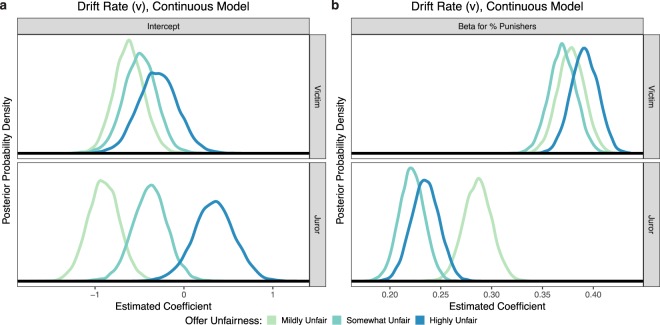
Effects of fairness violation severity on Victims’ and Jurors’ decisions to punish. (**a**) As indicated by the drift rate intercept, Victims do not distinguish between differing levels of offer unfairness, whereas Jurors do. (**b**) As shown by the drift rate betas, each additional punisher in the group provides stronger evidence that punishment should be favored, more so for Victims than Jurors.

While Jurors’ drift rates were found to increase as the proportion of punishers in the group increased, echoing the pattern found when Victims decided the outcome (Fig. 5), results also reveal interesting differences between Victims and Jurors in how strongly groups provide compelling evidence for punishment. Whereas Victims’ average *v* did not differ depending on offer unfairness, Jurors’ average *v* significantly increased as offers became increasingly unfair (both pairwise posterior *Ps* < 0.05). To further probe this relationship, we fit a variant model comparing Victims and Jurors, where the number of punishers within the group was treated as a continuous variable (see Supplementary Results). Results from this model indicate that the intercept of *v* does not significantly differ depending on offer unfairness for Victims (all posterior *Ps* > 0.1), but significantly increases as offers become more unfair for Jurors (all posterior *Ps* < 0.05; Fig. 6a). In other words, the degree of unfairness does not influence Victims’ valuation of punishment, whereas Jurors incorporate this information into their decisions, placing higher value on punishment as unfairness increases. Additionally, the regression betas for the number of punishers are significantly greater for Victims than Jurors (all posterior Ps < 0.001; Fig. 6b), meaning that each additional punisher in a group provides stronger evidence that punishment is valuable when one is an affected Victim than when one is an impartial Juror. These two patterns are also reflected in the behavioral data (Fig. 3e).

### Experiment 5: Crime judgments

Taken together, Experiments 1–4 demonstrate robust group influence on an individual’s punitive behavior, regardless of whether one is deciding as a Victim or Juror. However, our use of an economic game paradigm to precisely measure behavior leaves open an important question: is a group’s influence on an individual’s desire to punish limited to fairness violations, or does social influence act more generally upon a variety of moral violations? In order to answer this question, we performed a fifth experiment to examine whether a group can shift an individual’s judgments of how severely perpetrators should be punished for committing different types of crimes.

Subjects read short vignettes describing either a physical assault or a theft. The crime type (assault vs theft) was crossed with two levels of crime severity: high-intensity crimes involved the use of weapons, whereas low-intensity crimes did not. All vignettes featured an unambiguous perpetrator and victim. For subjects who completed the Victim condition, vignettes were written using second-person pronouns, and subjects were asked to imagine that they were the victim of the crime. The exact same vignettes were presented in the Juror condition, but instead of using second-person pronouns, the victims were instead unrelated strangers.

**Figure 7:**
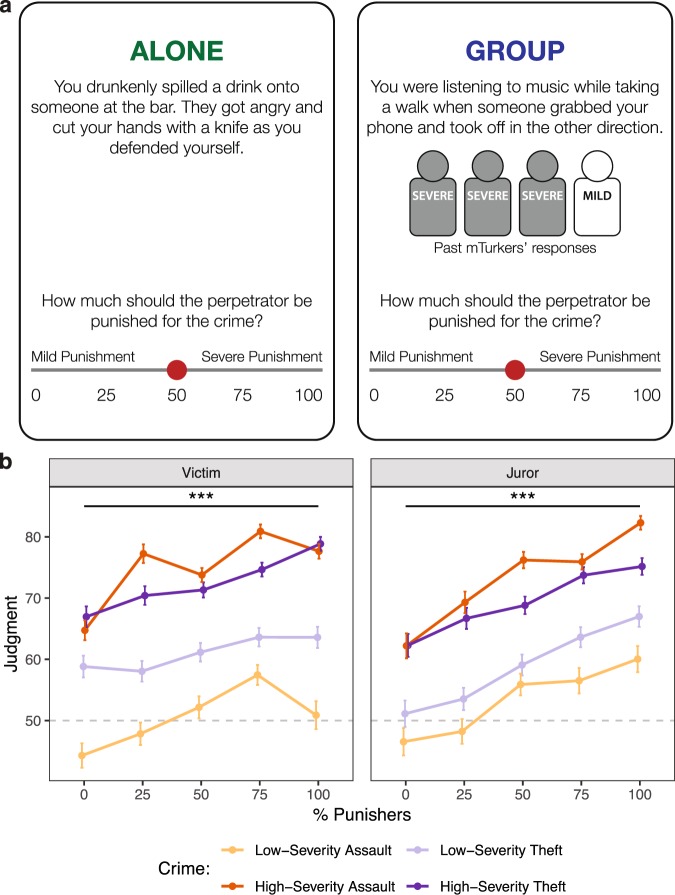
Change in punishment severity judgments for Victims and Jurors. (**a**) Schematic illustrating sample trials in which subjects are choosing alone or within a group. Vignettes were written in the second-party perspective in the Victim condition, and in the third-party perspective in the Juror condition. (**b**) Overall, the proportion of group members endorsing severe punishment increases subjects’ judgments for how severely a perpetrator should be punished for committing a crime. The midpoint of the scale is indicated using a grey dashed line. Error bars reflect ± 1SEM.

After reading the vignette, subjects were asked to rate how severely the perpetrator should be punished on a 100-point scale, where 0 corresponded to “Mild Punishment” and 100 corresponded to “Severe Punishment” (Fig. 7a). On Alone trials, subjects made their judgment in the absence of any social information. On Group trials, subjects were shown four icons that represented the responses of four past participants (though in reality, all responses were experimenter-generated to fully parameterize the decision space). The icons indicated the proportion of the group that had previously endorsed severe punishment as opposed to mild punishment.

To examine whether a group’s endorsement of punishment alters an individual’s judgments about the severity of punishment warranted by real-world crimes, we performed a linear mixed-effects regression, modeling judgments as an interaction between the proportion of punishers in the group (centered around 50% punishers), the type of crime (assault vs theft), and crime intensity (centered around medium intensity), and a covariate for subjects’ baseline preference for punishment (mean-centered and standardized). The overall pattern of results reveals that the proportion of group members endorsing severe punishment significantly modulates subjects’ judgments of how severely crimes should be punished (Fig. 7b), both when they are Victims (Table 5) and Jurors (Table 6). A formal comparison between Victims and Jurors further reveals that the overall conformity effect is significantly stronger for Jurors than for Victims (β = 2.06, *SE* = 0.69, *z* = 3.00, *df* = 224.00, *p* = 0.003), unlike our findings from Experiments 3–4. This divergence may be due to differences in moral decision-making when choices are hypothetical^41,42^, or may alternatively be due to the fact that the vignettes used in Experiment 5 involve moral transgressions that are more severe than the fairness violations in Experiments 1–4.

**Table 5:** Experiment 5: Punitive Judgments as a Victim.

*Punishment Judgment*_*i*,*t*_ = *β*_0_ + *β*_1_ *Proportion of Punishers*_*i*,*t*_ + *β*_2_ *Crime Type*_*i,t*_ + *β*_3_*Crime Severity*_*i,t*_ + *β*_4_ *Baseline Punishment*_*i*,*t*_				
Term	Estimate (*SE*)	*z*	*df*	*p*
Intercept	64.25 (0.60)	106.99	108.18	<0.001***
Proportion of Punishers	2.12 (0.41)	5.13	117.00	<0.001***
Crime Type (Reference category: assault)	−0.12 (0.60)	−0.20	104.85	0.840
Crime Severity	4.22 (0.51)	8.22	110.96	<0.001***
Baseline Punishment	14.10 (0.59)	23.71	91.00	<0.001***

Note: Terms are indexed by subject (*i*) and trial (*t*). The random effects structure is fully maximal: for each subject, the model includes a random intercept and random slopes for the proportion of punishers, crime type, crime severity, and baseline punishment.

*p < 0.05, **p < 0.01, ***p < 0.001.

**Table 6:** Punitive Judgments as a Juror.

*Punishment Judgment*_*i*,*t*_ = *β*_0_ + *β*_1_ *Proportion of Punishers*_*i*,*t*_ + *β*_2_ *Crime Type*_*i,t*_ + *β*_3_*Crime Severity*_*i*,*t*_ + *β*_4_ *Baseline Punishment*_*i*,*t*_				
Term	Estimate (*SE*)	*z*	*df*	*p*
Intercept	60.78 (0.65)	92.92	107.39	<0.001***
Proportion of Punishers	4.18 (0.56)	7.50	107.00	<0.001***
Crime Type (Reference category: assault)	2.30 (0.75)	3.06	98.46	0.003**
Crime Severity	2.54 (0.52)	4.83	96.56	<0.001***
Baseline Punishment	14.65 (0.76)	19.37	83.45	<0.001***

Note: Terms are indexed by subject (*i*) and trial (*t*). The random effects structure is fully maximal: for each subject, the model includes a random intercept and random slopes for the proportion of punishers, crime type, crime severity, and baseline punishment.

*p < 0.05, **p < 0.01, ***p < 0.001.

## Discussion

Groups often make better decisions than individuals by aggregating the knowledge, perspectives, and opinions of many people. This insight has led legal systems in many countries to use juries as fact-finders and arbiters of punishment^22^. Though juries are often able to use the ‘wisdom of the crowd’ to reach fair and judicious verdicts, groups can also produce conformist behavior, which may introduce biases into the decision-making process. Indeed, we find that subjects—Victims and Jurors alike—swiftly conform to groups’ punitive preferences, changing their own punishment behaviors by as much as 40 percentage points. Across all studies, this conformity effect scales linearly, such that individuals incrementally increase punishment as the proportion of punishers within the group grows larger. This indicates that individuals are integrating the group’s preference into their valuation of punishment, a conclusion that is additionally supported by the results of our computational model.

Though foundational theories of conformity have long invoked the metaphor of information processing^1,34^, little work has actually examined how individuals integrate others’ preferences into their own values as they are making choices. Therefore, by modeling decision-making as an information integration process, we extend a rich body of research on social influence by contributing three major insights into the cognitive dynamics that give rise to conformity. First, choosing in a group context causes individuals to become less cautious and more impulsive about their choices, making them especially susceptible to relinquishing moral responsibility and exhibiting conformist behavior. Second, being exposed to a group’s punitive preference amplifies how much an individual weighs evidence that punishment is a valuable mechanism for enacting justice. Third, groups are able to exert strong influence over individuals’ punitive behaviors regardless of individuals’ preexisting preferences for punishment. Specifically, despite Victims having an overall bias favoring compensatory responses over punitive ones (meaning that they do not seem to intrinsically value punishment), groups still can cause them to behave punitively.

These data suggest that punitive preferences are not held as sacred moral values. Instead, these results support an account in which decisions to punish a perpetrator are flexibly implemented, regardless of who is making those decisions and what the stakes of the choices are. By leveraging a computational model, we reveal two cognitive mechanisms explaining why both Victims and Jurors conform. First, both Victims and Jurors lowered their decision threshold when choosing in group contexts, which made them more susceptible to group influence because they were making less cautious and more impulsive choices. Second, as groups became increasingly punitive, they caused Victims and Jurors to value punishment more, as reflected by the growing drift rate.

The model, however, also reveals critical differences in how Victims and Jurors are influenced by groups. First, unlike Jurors, Victims are predisposed to a compensatory (non-punitive) response regardless of how unfair the offer was. This finding has clear implications for judicial bodies, as they may be inclined to punish perpetrators more than the victims themselves would desire^43^. Second, the rate at which Jurors integrate social information indicates that they weigh the group’s punitive preferences less than Victims. Third, Jurors, unlike Victims, actively incorporate information about the violation severity in their decision. This exquisite sensitivity of Jurors to the severity of violations is particularly interesting in the context of the principle of proportionality and legal debates on the role of victim impact statements on jury decisions. Therefore, although Jurors also conformed to the group’s punitive preference, they were more sensitive to the degree of the fairness violation and more resistant to use the group as a source of information about the value of punishment.

By demonstrating that groups can alter a victim or juror’s desire to punish a perpetrator, our data carry real-world implications for how mediators (such as ombudsmen and victim advocacy organizations) may influence their constituents’ preferences for justice restoration, and for how individuals serving on a jury may conform to fellow jurors’ punitive preferences. Indeed, our results point to a key vulnerability in decision-making that sets the stage for conformity, especially since our findings were not limited to fairness violations, but also extended to more serious crimes, such as assault and theft. Real-world social decisions depend on recurrent interactions between individuals that can result in cascading conformity effects. A small majority within the group could cause individuals to lower their decision threshold and conform to the majority opinion, thereby creating progressively larger majorities that provide stronger and stronger evidence for punishment’s value. Although it is difficult to obtain field data quantifying the extent to which conformity effects shift real jurors’ punitive inclinations, our results help to explain *why* conformity may be commonplace in jury deliberation^31^: we can readily and repeatedly induce the same conformist behaviors in naïve subjects, modify the rate at which subjects punish by manipulating the proportion of people endorsing punishment within a group, and explain how social influence impacts specific cognitive mechanisms involved in decisions to punish.

Of course, many questions still remain. For example, future work should examine how the strength of social influence changes when individuals repeatedly interact with the same perpetrators, especially when those perpetrators can learn to reform their behaviors over time. Given the theorized role of altruistic punishment in the enforcement and spread of prosocial behaviors like cooperation^8,20,21^, it is possible that conforming to a group’s punitive preferences might cease once an individual perceives that a perpetrator has been successfully rehabilitated. Repeated interactions with perpetrators will also allow researchers to build upon literature characterizing social influence and attitude change as learning processes^6,44^. This may reveal that, depending on how much decision ambiguity they are able to resolve^45^, individuals modulate their willingness to conform to a group by drawing upon previously-learned social knowledge. While real-world punishment decisions are undoubtedly informed by a variety of factors, our experiments reveal the potency of groups in swaying an individual’s punitive preferences by providing individuals with evidence that punishment is a socially-valued method of restoring justice.

## Methods

### Subjects

In Experiments 1–4, 176 subjects were recruited from Brown University and the surrounding community. All methods were approved by the Brown University Institutional Review Board, and carried out according to the approved procedures. Informed consent was also obtained from each subject in a manner approved by Brown University’s Institutional Review Board. Subjects were paid $10/hr or received partial course credit for participation. Subjects in Experiments 1–3 earned an additional monetary bonus (up to $9 in Experiments 1–2, and up to $9.90 in Experiment 3) depending on the choices they made. All study sessions lasted one hour. Based on extant research, we aimed to achieve a final sample size of N = 40 for all in-lab experiments^44^. To achieve this target sample size, we made an *a priori* decision in Experiments 1–2 to recruit additional subjects in case there were any potential missing data resulting from excluding highly-suspicious subjects (a routine feature of experiments using social deception). Based on a scale where 1 = not suspicious at all and 6 = very suspicious, high suspicion was operationalized *a priori* as a rating of 5 or higher. These additional subjects were recruited prior to analyzing any data in an effort to minimize false positives from inflating researcher degrees of freedom.

In Experiment 1 (Last Decider), we ran 42 subjects; one subject’s data were lost due to a technical error, leaving a final sample of 41 subjects (32 female; 25 non-White or mixed-race; mean age = 19.85, SD ± 1.81). One subject expressed high levels of suspicion (mean suspicion = 2.39, SD ± 1.09). In Experiment 2 (First Decider), we recruited 48 subjects (35 female; 23 non-White or mixed-race; mean age = 19.31, SD ± 1.45). Five subjects reported suspicion (mean suspicion = 2.63, SD ± 1.30), and three subjects indicated some confusion about the task during the debrief. Because subjects’ suspicion did not have a significant effect when included as a covariate in the regression models (Last Decider *p* = 0.660, First Decider *p* = ﻿0.994), we include their data in our analysis. Because our results were robust to subjects’ suspicion in Experiments 1–2, we decided not to recruit additional subjects to potentially replace suspicious subjects in Experiments 3–4.

In Experiment 3 (Victim DDM), we ran 41 subjects. Due to a technical error, only partial data is available from one subject, which we included in our analysis. Demographic information about this subject is unavailable. Of the 40 remaining subjects, 31 were female; 20 were non-White or mixed-race, and the mean age was 20.95, SD ± 2.49. Four subjects reported being suspicious (mean suspicion = 2.38, SD ± 1.27). In Experiment 4 (Juror DDM), we ran 45 subjects. Due to a technical error, only partial data is available for four subjects. Partial data is also available for an additional subject who unexpectedly became ill during the experimental session. We included only partial data in which subjects completed more than 100 trials in our final analysis, leaving a final sample of 43 subjects (32 female; 24 non-White or mixed-race; mean age = 19.72, SD ± 3.36). Seven subjects reported some suspicion (mean suspicion = 2.74, SD ± 1.36). Once again, subjects’ suspicion did not have a significant effect when included as a covariate in the regression models (Victim *p* = ﻿0.274, Juror *p* = 0.907), and thus our analyses retains all subjects’ data.

In Experiment 5 (Crime Judgments), we recruited 300 subjects from Amazon Mechanical Turk, half of whom participated in the Victim condition, and the half in the Juror condition. Informed consent was obtained from each subject in a manner approved by Brown University’s Institutional Review Board. Subjects were paid $5/hr. Study sessions lasted approximately one hour. The subject pool was restricted to IP addresses from the United States, and to workers who had completed ≥50 HITs with ≥95% HIT approval rate. Due to concerns about data quality on mTurk^46^, subjects were required to provide a free-response answer to the open-ended prompt: “In your own words, please define the word crime. There is no single correct answer, so please provide a definition in your own words.” We excluded subjects who had provided off-topic responses or who had (to the best of our knowledge) copy-pasted responses from sources such as Google and Wikipedia. As a consequence, 32 responses were dropped from the Victim dataset and 42 responses were dropped from the Juror dataset, leaving a final sample of 118 subjects in the Victim condition (44 female; 20 non-White or mixed-race; mean age = 33.98, SD ± 7.97) and 108 subjects in the Juror condition (43 female; 17 non-White or mixed-race; mean age = 32.84, SD ± 7.97). In the Victim condition, 47 subjects reported some suspicion (mean suspicion = 3.81, SD ± 1.53), and in the Juror condition, 44 subjects reported some suspicion (mean suspicion = 4.05, SD ± 1.51). Subjects’ suspicion did not have a significant effect when included as a covariate in the regression models (Victim *p* = 0.901, Juror *p* = 0.335).

### Experimental protocol

#### Experiments 1–2: Last Decider and First Decider

Although subjects were led to believe that they would be randomly assigned to the role of Player A or Player B once at the beginning of the experiment, they were always assigned to be Player B. Subjects believed that the players they were interacting with were past participants who had previously come into the lab and completed the experiment. In reality, however, all responses from other players were computer-generated to fully parameterize the decision space. Subjects were told that we had previously collected the mailing addresses of past participants, that we would randomly select one trial to pay out at the end of the study, and that we would enact their choices as Player B on that trial by mailing a check to past participants. Accounting for the possibility that subjects might infer strategic motives behind Player A offers, payout at the end of the study was implemented probabilistically such that Player A’s original offer was enacted half of the time and Player B’s decision was enacted half of the time (see Supplementary Methods). Player As were represented by photographs of faces in order to enhance believability of the social deception manipulation. Photographs of fictitious partners were drawn from the Chicago Face Database and the MR2 database^47,48^. In order to avoid confounding effects of race and gender, we only used images of white male faces. Further details can be found in the Supplementary Methods.

In the Solo Phase of the task, subjects completed two trials for each fairness level for a total of eight randomly-presented trials. In the Group Phase of the task, subjects shared the role of Player B with four other players, and made a collective decision based on a simple majority. Though subjects were told that the sequence in which they decided in the Group Phase would be randomly determined once at the beginning of the experiment, the sequence was fixed such that subjects in Experiment 1 were always last to decide (Fig. 1b), and subjects in Experiment 2 were always first to decide (Fig. 1c). The number of Reverse responses from other Player Bs was parametrically varied such that zero to four partners reversed on each trial out of a fixed total of four partners, and every permutation was presented. Responses to highly unfair offers (8/2 and 9/1 splits to Player B) were sampled at twice the frequency as responses to mildly unfair offers (6/4 and 7/3 splits). In total, subjects completed 96 trials in the Group Phase of the study. Subjects had up to eight seconds to make a choice, after which the computer automatically selected the Accept option and moved on to the next trial. Prior to beginning either the Solo Phase or the Group Phase, subjects completed verbal quizzes to ensure that they understood all the rules of the game and were given an opportunity to ask any questions before beginning the task. After completing both the Solo and Group Phases of the experiment, subjects completed a demographic survey, were probed for suspicion that their partners might not be real, and were debriefed.

#### Experiments 3–4: Victim and Juror DDM

Experiment 3 (Victim DDM) closely resembles Experiment 1 (Last Decider) with the following major differences. First, subjects could only choose between the Compensation and Reverse options; response key mapping was counterbalanced across subjects and remained consistent across trials and task phases for each subject. Second, Alone trials were intermixed with Group trials. Subjects completed a total of 420 trials in the Group Phase, of which 375 were Group trials and 45 were Alone trials. Third, offers from Player As were drawn from bounded uniform distributions in 10¢ increments, such that Mildly Unfair offers ranged from $3.70-$4.90, Somewhat Unfair offers ranged from $1.90-$3.10, and Highly Unfair offers ranged from $0.10-$1.30. Once a subject made a response, they were shown a feedback screen showing the outcome of their choice (i.e. the amount of money Player A and Player B would receive based on Player B’s decision). Fourth, photographs of group members were replaced with images of blue dots to avoid potential confounds associated with the use of face stimuli. Fifth, subjects were presented with the entire group’s votes simultaneously in order to capture the decision-making process in its entirety. Finally, all subjects saw the same predetermined fully-randomized trial sequence.

Because DDM is sensitive to reaction time outliers, we excluded trials in which reaction times were faster than 0.3 s or slower than 5 s. This consisted of 9.88% of all trials in Experiment 3 and 4.74% of trials in Experiment 4. The 0.3 s cutoff was chosen as a conservative threshold for fast outliers based on best practices in the drift diffusion modeling literature^49^, and also given that even the simplest perceptual discrimination decisions are rarely made faster than 0.3 s. Similarly, given that DDMs have been best characterized for relatively fast decisions of 1–1.5s^37^, we chose to threshold slow outliers at a conservative cutoff of 5 s, which excluded a small number of trials^49^. For consistency, both our behavioral and DDM analyses were performed on this clipped data. To further guard DDM estimates against outliers, our model assumed 5% of trials to be distributed from a uniform distribution, as opposed to the DDM likelihood. This procedure is specifically recommended by the HDDM developers as a best practice^39^. One subject in Experiment 3 was excluded from the Highly Unfair analysis because their data contained missing cells. Mean *v* and *a* for each fairness type was calculated by adding the regression coefficient for each number of punishers (zero to four punishers) compared to deciding Alone, which we treated as the intercept.

HDDM uses Markov Chain Monte Carlo sampling methods to generate posterior distributions for estimated parameters; chain convergence was assessed using the Gelman-Rubin statistic (all scale reduction factors <1.1), and we obtained 10,000 samples of each posterior distribution to obtain smooth parameter estimates. We tested multiple models allowing different parameters to vary according to the number of punishers within the group. The model parameters reported are from the best-fitting model that most faithfully matches the design of our task. Model selection was performed using the Deviance Information Criterion (DIC), which assesses goodness of model fit while penalizing for model complexity. Goodness of fit was also assessed using a posterior predictive check. To ensure that HDDM is capable of accurately estimating our model’s parameters, we also performed parameter recovery simulations using the parameters that were estimated for our model. Details are discussed in the Supplementary Methods.

The procedure for Experiment 4 (Juror DDM) was identical to that of Experiment 3, with two key differences. First, because subjects were making third-party punishment decisions, we informed subjects that their choices would not affect their own monetary outcomes and required subjects to recount this information in a verbal quiz before beginning the task. Second, each subject saw a unique and fully-randomized trial sequence. The procedure used to analyze DDM results was identical to that of Experiment 3. The best-fitting model from Experiment 3 was used to estimate parameters for Experiment 4 (see Supplementary Information for details).

#### Experiment 5: Crime judgments

Alone and Group trials were intermixed to avoid potential ordering confounds. Subjects completed two trials for each unique stimulus type for a total of 48 trials. Subjects were informed that group members endorsing “mild punishment” had judged the appropriate punishment as being under 50 on a 100-point scale, while group members endorsing “severe punishment” had judged the appropriate punishment as being above 50. All vignettes used in the study are available in the Supplementary Methods.

## Supplementary information

Supplementary Information - Crowdsourcing punishment: individuals reference group preferences to inform their own punitive decisions

## Data Availability

The data that support the findings of this study are available online at the Open Science Framework at the following URL: https://osf.io/8ka47/.

## References

[1] Asch SE (1956). Studies of independence and conformity: I. A minority of one against a unanimous majority. Psychological Monographs: General and Applied.

[2] Cialdini RB (2003). Crafting Normative Messages to Protect the Environment. Current Directions in Psychological Science.

[3] Koban L, Wager TD (2016). Beyond conformity: Social influences on pain reports and physiology. Emotion.

[4] Berns GS, Capra CM, Moore S, Noussair C (2010). Neural mechanisms of the influence of popularity on adolescent ratings of music. NeuroImage.

[5] Campbell-Meiklejohn DK, Bach DR, Roepstorff A, Dolan RJ, Frith CD (2010). How the Opinion of Others Affects Our Valuation of Objects. Current Biology.

[6] Klucharev V, Hytönen K, Rijpkema M, Smidts A, Fernández G (2009). Reinforcement Learning Signal Predicts Social Conformity. Neuron.

[7] Zaki J, Schirmer J, Mitchell JP (2011). Social Influence Modulates the Neural Computation of Value. Psychological Science.

[8] Fowler JH, Christakis NA (2010). Cooperative behavior cascades in human social networks. Proceedings of the National Academy of Sciences.

[9] Frey BS, Meier S (2004). Social Comparisons and Pro-Social Behavior: Testing “Conditional Cooperation” in a Field Experiment. The American Economic Review.

[10] Nook EC, Ong DC, Morelli SA, Mitchell JP, Zaki J (2016). Prosocial Conformity: Prosocial Norms Generalize Across Behavior and Empathy. Personality and Social Psychology Bulletin.

[11] Tetlock PE (2003). Thinking the unthinkable: sacred values and taboo cognitions. Trends in Cognitive Sciences.

[12] Peysakhovich, A., Nowak, M. A. & Rand, D. G. Humans display a ‘cooperative phenotype’ that is domain general and temporally stable. **5**, 4939, 10.1038/ncomms5939 (2014). 10.1038/ncomms5939 25225950

[13] Haidt J (2001). The emotional dog and its rational tail: A social intuitionist approach to moral judgment. Psychological Review.

[14] Van Bavel JJ, Packer DJ, Haas IJ, Cunningham WA (2012). The Importance of Moral Construal: Moral versus Non-Moral Construal Elicits Faster, More Extreme, Universal Evaluations of the Same Actions. PLOS ONE.

[15] Luttrell A, Petty RE, Briñol P, Wagner BC (2016). Making it moral: Merely labeling an attitude as moral increases its strength. Journal of Experimental Social Psychology.

[16] Ryan TJ (2017). No Compromise: Political Consequences of Moralized Attitudes. American Journal of Political Science.

[17] Ginges J, Atran S, Medin D, Shikaki K (2007). Sacred bounds on rational resolution of violent political conflict. Proceedings of the National Academy of Sciences.

[18] Strohminger N, Nichols S (2014). The essential moral self. Cognition.

[19] Fehr E, Fischbacher U (2004). Third-party punishment and social norms. Evolution and Human Behavior.

[20] Fehr E, Gächter S (2002). Altruistic punishment in humans. Nature.

[21] Henrich J (2006). Costly Punishment Across Human Societies. Science.

[22] Hans VP (2008). Jury Systems Around the World. Annual Review of Law and Social Science.

[23] Paternoster RAY, Deise J (2011). A heavy thumb on the scale: The effect of victim impact evidence on capital decision making. Criminology.

[24] North, A. Why women are worried about #MeToo. *Vox* https://www.vox.com/2018/4/5/17157240/me-too-movement-sexual-harassment-aziz-ansari-accusation (2018).

[25] Pascoe D (2016). Is Diya a Form of Clemency?. Boston University International Law Journal.

[26] Wald, P. M. Dealing with Witnesses in War Crime Trials: Lessons from the Yugoslav Tribunal. *Yale Human Rights & Development Law Journal* **5** (2002).

[27] Keller, J. Anti-Government Unrest and American Vigilantism. *The Atlantic* https://www.theatlantic.com/politics/archive/2010/03/anti-government-unrest-and-american-vigilantism/38229/ (2010).

[28] Bohstedt, J. In *The dynamics of aggression: Biological and social processes in dyads and groups* (eds Potegal, M. & Knutson, J. F.) 257–306 (Erlbaum Hillsdale, NJ, 1994).

[29] Mooijman M, Hoover J, Lin Y, Ji H, Dehghani M (2018). Moralization in social networks and the emergence of violence during protests. Nature Human Behaviour.

[30] Maccoun RJ (1989). Experimental Research on Jury Decision-Making. Science.

[31] Waters NL, Hans VP (2009). A Jury of One: Opinion Formation, Conformity, and Dissent on Juries. Journal of Empirical Legal Studies.

[32] Irazola, S., Williamson, E., Stricker, J. & Niedzwiecki, E. Study of Victim Experiences of Wrongful Conviction. (ICF International, 2013).

[33] FeldmanHall, O., Sokol-Hessner, P., Van Bavel, J. J. & Phelps, E. A. Fairness violations elicit greater punishment on behalf of another than for oneself. **5**, 5306, 10.1038/ncomms6306 (2014). 10.1038/ncomms6306 PMC4266485 25350814

[34] Deutsch M, Gerard HB (1955). A study of normative and informational social influences upon individual judgment. The Journal of Abnormal and Social Psychology.

[35] Germar M, Schlemmer A, Krug K, Voss A, Mojzisch A (2013). Social Influence and Perceptual Decision Making. Personality and Social Psychology Bulletin.

[36] Hutcherson CA, Bushong B, Rangel A (2015). A Neurocomputational Model of Altruistic Choice and Its Implications. Neuron.

[37] Ratcliff R, McKoon G (2007). The Diffusion Decision Model: Theory and Data for Two-Choice Decision Tasks. Neural Computation.

[38] Voss A, Rothermund K, Voss J (2004). Interpreting the parameters of the diffusion model: An empirical validation. Memory & Cognition.

[39] Wiecki T, Sofer I, Frank M (2013). HDDM: Hierarchical Bayesian estimation of the Drift-Diffusion Model in Python. Frontiers in Neuroinformatics.

[40] Kruschke John K. (2013). Bayesian estimation supersedes the t test. Journal of Experimental Psychology: General.

[41] FeldmanHall O (2012). What we say and what we do: The relationship between real and hypothetical moral choices. Cognition.

[42] FeldmanHall O (2012). Differential neural circuitry and self-interest in real vs hypothetical moral decisions. Social Cognitive and Affective Neuroscience.

[43] Alliance for Safety and Justice. *Crime Survivors Speak: The First-Ever National Survey of Victims’ Views on Safety and Justice*, https://allianceforsafetyandjustice.org/crimesurvivorsspeak/ (2016).

[44] FeldmanHall Oriel, Otto A. Ross, Phelps Elizabeth A. (2018). Learning moral values: Another’s desire to punish enhances one’s own punitive behavior. Journal of Experimental Psychology: General.

[45] FeldmanHall O, Shenhav A (2019). Resolving uncertainty in a social world. Nature Human Behaviour.

[46] Dennis, S. A., Goodson, B. M. & Pearson, C. Virtual private servers and the limitations of IP-based screening procedures: Lessons from the MTurk quality crisis of 2018. *SSRN*, 10.2139/ssrn.3233954 (2018).

[47] Ma DS, Correll J, Wittenbrink B (2015). The Chicago face database: A free stimulus set of faces and norming data. Behavior Research Methods.

[48] Strohminger N (2016). The MR2: A multi-racial, mega-resolution database of facial stimuli. Behavior Research Methods.

[49] Ratcliff R, Tuerlinckx F (2002). Estimating parameters of the diffusion model: approaches to dealing with contaminant reaction times and parameter variability. Psychonomic bulletin & review.

[50] Barr DJ, Levy R, Scheepers C, Tily HJ (2013). Random effects structure for confirmatory hypothesis testing: Keep it maximal. Journal of Memory and Language.

