# Supplementary material for: Crowdsourcing punishment: Individuals reference group preferences to inform their own punitive decisions
Source: Sci Rep. 2019 Aug 12;9:11625. doi: 10.1038/s41598-019-48050-2 (PMC6690944; doi:10.1038/s41598-019-48050-2)
Supplement: Supplementary file 1 — Supplementary Information - Crowdsourcing punishment: individuals reference group preferences to inform their own punitive decisions [file 41598_2019_48050_MOESM1_ESM.docx]

**SUPPLEMENT – Crowdsourcing punishment: individuals reference group preferences to inform their own punitive decisions**

Jae-Young Son^1^, Apoorva Bhandari^1^, & Oriel FeldmanHall^1, 2^

^1^Department of Cognitive, Linguistic, and Psychological Sciences, Brown University, Providence, RI 02906

^2^Carney Institute for Brain Science, Brown University, Providence, RI 02906

Corresponding Author:

Oriel FeldmanHall

Brown University

190 Thayer St. Providence, RI 02906

oriel.feldmanhall@brown.edu

**SUPPLEMENTARY RESULTS**

**EXPERIMENTS 1 AND 2: LAST DECIDER AND FIRST DECIDER**

*Punishment in the Solo Phase.* To examine whether subjects were sensitive to the unfairness of Player A’s offers in the Solo Phase, we performed a mixed-effects logistic regression predicting punishment decisions by offer unfairness. The model included a subject-specific random intercept and random slope for offer unfairness. Results indicate that offer unfairness did not have a significant effect on punishment rates in Experiment 1 (Last Decider; β=﻿0.03, *SE*=0.17, *z*=﻿0.19, *p*=0.850), and did have a significant effect on punishment rates in Experiment 2 (First Decider; β=﻿0.45, *SE*=0.17, *z*=﻿2.63, *p*=0.009).

***Figure S1 | Punishment rates in the Solo Phase of Experiments 1 (Last Decider) and 2 (First Decider).*** *Error bars reflect ± 1 SEM.*

*Model-fitting and selection procedure.* Given previous work illustrating that unfairness does not seem to have a systematic effect when learning about another’s punitive desires (FeldmanHall, Otto, & Phelps, 2018), we were agnostic as to whether conformist behavior would scale with the severity of the violation. Accordingly, we performed three mixed-effects logistic regressions that tested various roles for unfairness during the conformity processes. In the first regression, we specified an additive model characterizing punishment as a linear combination of the proportion of punishers. In the second regression, we specified an interactive model including an extra term according for an interaction between the proportion of punishers and offer unfairness. Finally, we performed a third regression excluding offer unfairness to test the possibility that offer unfairness did not affect punishment decisions. All models included a random intercept for each subject, as well as a random slope for the proportion of punishers. A random slope for offer unfairness could not be estimated in the additive and interactive models due to convergence failure and was therefore excluded (Barr, Levy, Scheepers, & Tily, 2013).

All models were evaluated in R using the lme4 package (Bates, Mächler, Bolker, & Walker, 2015), and p-values were calculated using the lmerTest package (Kuznetsova, Brockhoff, & Christensen, 2017). To ensure that estimates were not inflated due to multicollinearity between predictors, we calculated the Variance Inflation Factor (VIF) for all models and ensured that all VIFs were below 5, indicating low multicollinearity (Sheather, 2009).

Model selection was performed using the likelihood ratio test, which provides a quantitative goodness-of-fit measure for two competing models, and can therefore be used to parsimoniously adjudicate between them. For both the Last Decider and First Decider experiments, the additive model was found to have a superior goodness-of-fit to the simple model, and the more complex interactive model did not further improve the goodness-of-fit. Therefore, we report the best-fitting model (additive) in the main text.

***Figure S2 | Predicted punishment rates in Experiments 1 (Last Decider) and 2 (First Decider).*** *Preference for punishment increases with the proportion of punishers in both the Last Decider and First Decider experiments. This effect is reduced, but remains significant, when subjects are observing the moral preferences of past groups. The y-axes are drawn at different scales for the two experiments. Shaded areas reflect ± 1 SEM.*

*Alternative models of social influence in Experiment 2 (First Decider).* In the First Decider experiment, subjects only had direct access to the unfairness of Player A’s offer on the current trial (*t*). However, we predicted that individuals’ punitive decisions would still be susceptible to social influence from past groups. Specifically, we predicted that subjects would refer back to the last trial where Player A’s offer matched that of the current trial *t*. For convenience, we refer to this strategy as *t-N*, where *t* refers to the current trial and *N* refers to the number of trials that have elapsed since the most recent match. We report this operationalization of social influence in the main text. However, it is not the only plausible model of influence. It may instead have been the case that subjects simply drew upon what the group’s preference was on the immediately preceding trial (*t-1*), akin to a priming effect.

To test the *t-1* operationalization, we redefined the proportion of punishers on trial *t* as the group’s punitive preference on the preceding trial. The regression model specification was otherwise identical to that of Experiment 2. Results indicate that the *t-1* model is a less good fit for the data (BIC=﻿﻿2250.25) than the *t-N* model (BIC=﻿2156.31). Additionally, the overall pattern of results for the *t-1* model is qualitatively similar to those of the *t-N* model reported in the main text (Table S1).

***Table S1 | Priming model of social influence (t-1) in Experiment 2 (First Decider)***

| ${Punishment}_{i,t}= \beta_{0}+\beta_{1}{Proportion of Punishers}_{i,t}+ \beta_{2}{Offer Unfairness}_{i,t}+\beta_{3}{Baseline Punishment}_{i,t}$ | | | |
| --- | --- | --- | --- |
| Term | Estimate (*SE*) | *z* | *p* |
| Intercept | ﻿-2.78 (0.66) | -4.22 | < 0.001 *** |
| Proportion of Punishers | 0.32 (0.14) | 2.25 | ﻿0.025 * |
| Offer Unfairness | 0.46 (0.04) | 11.82 | < 0.001 *** |
| Baseline Punishment | 1.58 (0.56) | 2.81 | 0.005 ** |
| Note: Terms are indexed by subject (*i*) and trial (*t*). For each subject, the model includes a random intercept and a random slope for the proportion of punishers.  * *p* < 0.05, ** *p* < 0.01, *** *p* < 0.001 | | | |

*Effects of trial history in Experiment 2 (First Decider).* Finally, we performed an exploratory analysis to determine whether the number of trials elapsed since the most recent match in offer unfairness (i.e., the value of *N* in *t-N*) impacts punishment. To do this, we performed two mixed-effects logistic regressions. For the additive variant, the model specification was similar to Experiment 2, with the addition of a predictor for the number of trials that had elapsed since the last trial matching the offer unfairness of the current trial (*N*). For the interactive variant, the model additionally included an interaction term between the proportion of punishers and trial history. Including subject-specific random slopes for baseline punitive preference caused singular fits, and therefore this term was not estimated. Results suggest that trial history does not modulate punishment in either the additive model (β=﻿-0.01, *SE*=0.02, *z*=﻿-0.87, *p*=0.383), nor the interactive model (β=﻿-0.01, *SE*=0.02, *z*=﻿-0.83, *p*=0.407). Furthermore, there is no evidence for an interaction between the proportion of punishers and trial history (β=﻿-0.02, *SE*=0.02, *z*=﻿-1.41, *p*=0.157).

**EXPERIMENTS 3 AND 4: DRIFT DIFFUSION MODEL OF CONFORMITY AS A VICTIM OR JUROR**

*Punishment in Alone trials.* To examine whether subjects were sensitive to the unfairness of Player A’s offers in Alone trials, we performed a mixed-effects logistic regression predicting punishment decisions by offer unfairness. The model included a subject-specific random intercept and random slope for offer unfairness. Results indicate that offer unfairness did not have a significant effect on punishment rates in Experiment 3 (Victim; β=﻿0.33, *SE*=0.29, *z*=﻿1.15, *p*=0.250), and did have a significant effect on punishment rates in Experiment 4 (Juror; β=﻿0.58, *SE*=0.17, *z*=﻿3.44, *p* < 0.001).

***Figure S3 | Punishment rates in the Alone trials of Experiments 3-4.*** *Error bars reflect ± 1 SEM.*

*Behavioral model-fitting and selection procedure.* The procedure for fitting and selecting regression models in Experiments 3-4 was identical to Experiments 1-2. For both the Victim and Juror experiments, the additive model was found to have a superior goodness-of-fit to the simple model, and the more complex interactive model did not further improve the goodness-of-fit. Therefore, we report the best-fitting model (additive) in the main text.

***Figure S4 | Predicted punishment rates in Experiments 3 (Victim) and 4 (Juror).*** *Preference for punishment increases with the proportion of punishers in both the Victim and Juror experiments. Shaded areas reflect ± 1 SEM.*

*Descriptive statistics of reaction times.* In the main text, we were primarily interested in interpreting the estimated parameters from our model, rather than making inferences from model-free reaction time (RT) distributions and choice proportions. For thoroughness, descriptive statistics of raw RTs and punishment rates are reported here. After clipping RTs faster than 0.3 seconds and longer than 5 seconds, one subject did not have any remaining data for Highly Unfair offers; that subject’s data are omitted from all relevant behavioral analyses to ensure that the reported behavioral findings reflect the analysis of our model as closely as possible. As a general qualitative trend, we observe that as the proportion of punishers increases, decisions to Reverse get faster, while decisions to Compensate get slower (Figure S5; mean RTs are also numerically reported in Table S2).

***Figure S5 | Reaction times in Experiments 3 (Victim) and 4 (Juror).*** ***A)*** *Reaction time (RT) density for Victims. Rows indicate the proportion of punishers within the group.* ***B)*** *RT density for Jurors.* ***C)*** *Mean RTs for Victims. The letter “A” on the x-axis stands for Alone trials. Error bars reflect ± 1 SEM.* ***D)*** *Mean RTs for Jurors.*

***Table S2 | Mean RTs in Experiments 3-4 (Victim and Juror DDM).*** *RTs reported in seconds.*

| **Experiment** | **Offer Unfairness** | **% Punishers** | **Mean Compensate (SD)** | **Mean Reverse (SD)** |
| --- | --- | --- | --- | --- |
| Victim DDM | Mildly Unfair | Alone | 1.16 (0.74) | 1.40 (0.59) |
|  |  | 0% | 0.95 (0.56) | 1.53 (0.86) |
|  |  | 25% | 0.95 (0.53) | 1.27 (0.68) |
|  |  | 50% | 1.09 (0.57) | 1.23 (0.69) |
|  |  | 75% | 1.21 (0.76) | 1.40 (0.86) |
|  |  | 100% | 1.28 (0.81) | 1.27 (0.61) |
|  | Somewhat Unfair | Alone | 1.17 (0.58) | 1.37 (0.56) |
|  |  | 0% | 1.02 (0.57) | 1.35 (0.69) |
|  |  | 25% | 1.09 (0.74) | 1.52 (0.76) |
|  |  | 50% | 1.23 (0.72) | 1.43 (0.64) |
|  |  | 75% | 1.26 (0.81) | 1.25 (0.68) |
|  |  | 100% | 1.29 (0.83) | 1.06 (0.46) |
|  | Highly Unfair | Alone | 1.05 (0.75) | 1.08 (0.45) |
|  |  | 0% | 0.97 (0.55) | 1.38 (0.86) |
|  |  | 25% | 0.99 (0.53) | 1.34 (0.65) |
|  |  | 50% | 1.04 (0.60) | 1.30 (0.61) |
|  |  | 75% | 1.02 (0.66) | 1.17 (0.62) |
|  |  | 100% | 1.09 (0.65) | 1.16 (0.67) |
| Juror DDM | Mildly Unfair | Alone | 1.03 (0.46) | 1.38 (0.85) |
|  |  | 0% | 0.88 (0.45) | 1.21 (0.53) |
|  |  | 25% | 0.86 (0.32) | 1.38 (0.98) |
|  |  | 50% | 0.98 (0.46) | 1.17 (0.88) |
|  |  | 75% | 1.03 (0.53) | 1.12 (0.61) |
|  |  | 100% | 1.04 (0.51) | 1.00 (0.56) |
|  | Somewhat Unfair | Alone | 1.07 (0.51) | 1.27 (0.51) |
|  |  | 0% | 0.95 (0.50) | 1.43 (0.65) |
|  |  | 25% | 1.02 (0.58) | 1.49 (0.85) |
|  |  | 50% | 1.10 (0.54) | 1.38 (0.74) |
|  |  | 75% | 1.11 (0.61) | 1.16 (0.51) |
|  |  | 100% | 1.17 (0.49) | 1.19 (0.67) |
|  | Highly Unfair | Alone | 0.96 (0.49) | 1.20 (0.58) |
|  |  | 0% | 0.93 (0.51) | 1.07 (0.41) |
|  |  | 25% | 0.81 (0.32) | 1.18 (0.61) |
|  |  | 50% | 1.09 (0.68) | 1.14 (0.41) |
|  |  | 75% | 1.13 (0.77) | 0.93 (0.33) |
|  |  | 100% | 0.95 (0.43) | 1.09 (0.67) |

*HDDM model selection.* We used HDDM’s built-in regression function to construct linear models that allow DDM parameters to vary by the proportion of punishers on each trial (Wiecki, Sofer, & Frank, 2013). We predicted that the drift rate (*v*) and threshold (*a*) would meaningfully vary by the proportion of punishers within a group. To test this in Experiment 3 (Victim DDM), we ran multiple types of models in which different permutations of DDM parameters were permitted to vary by the proportion of punishers within a group (Table S3). Goodness of fit was assessed using the Deviance Information Criterion (DIC)—a variant of statistics such as the Akaike Information Criterion (AIC) and the Bayesian Information Criterion (BIC)—which is especially useful when a Markov Chain Monte Carlo (MCMC) is used to obtain the posterior distribution of a model. A comparison of alternative models’ DICs demonstrates that the model we report is the best-fitting model after penalizing for model complexity. We would like to note that it is psychologically implausible for bias (*z*) to vary by the proportion of punishers; *z* is set prior to observing evidence, and we treat the proportion of punishers as evidence. We report the DIC from models in which *z* is permitted to vary by the proportion of punishers only to provide a sense for how our reported model performs against implausible “null” models with comparable model complexity. As predicted, the model with the lowest DIC is the one in which *v* and *a* are permitted to vary by the proportion of punishers (Table S3, Model Ex3-4a).

To test whether drift rate followed a linear trend in our best-fitting model (i.e. Model Ex3-4a), we ran an additional exploratory regression (Model Ex3-4b), in which we excluded all Alone trials from analysis, and in which the number of punishers was entered as a continuous variable when estimating *v* and a categorical variable when estimating *a* (using “two punishers out of a group of four people” as the reference category). The DIC for this model suggests that the linear trend explains our data well, as we observe a better model fit even with less data used to fit the model. Because we were interested in modeling subject responses to Alone trials, we report the second-best-fitting model in the main manuscript. However, we would like to note that both models produce similar patterns of results that we interpret identically.

For Experiment 4 (Juror DDM), we were primarily interested in comparing the estimated parameters against those obtained in Experiment 3 (Victim DDM). Therefore, we only estimated models in which the parameters *v* and *a* were permitted to vary by the number of punishers in the group, to match models Ex3-4a and Ex3-4b from Experiment 3. Model fits are reported in Table S3.

***Table S3 | Model comparison in Experiments 3-4.*** *Ex3 refers to models from Experiment 3, and Ex4 refers to models from Experiment 4. Smaller DIC values indicate better model fit.*

| **Model** | **Parameters that vary by # of punishers** | **Mildly Unfair DIC** | **Somewhat Unfair DIC** | **Highly Unfair DIC** | **Average DIC** |
| --- | --- | --- | --- | --- | --- |
| Ex3-1 | *Drift rate (v)* | 7129.49 | 7698.83 | 6314.64 | 7047.65 |
| Ex3-2 | *Threshold (a)* | 7975.06 | 8366.61 | 7160.79 | 7834.15 |
| Ex3-3 | *Bias (z)* | 7774.43 | 8281.25 | 6874.80 | 7643.49 |
| Ex3-4a | *v*, *a* (categorical *v*) | 7042.49 | 7566.66 | 6261.27 | 6956.81 * |
| Ex3-4b | *v*, *a* (continuous *v*) | 6237.51 | 6652.25 | 5729.34 | 6206.37 ** |
| Ex3-5 | *v*, *z* | 7125.45 | 7708.01 | 6319.61 | 7051.02 |
| Ex3-6 | *a*, *z* | 7670.37 | 8138.10 | 6827.61 | 7545.36 |
| Ex3-7 | *v*, *a*, *z* | 7044.88 | 7568.58 | 6259.50 | 6957.66 |
| Ex4-1a | *v*, *a* (categorical *v*) | 6363.62 | 9316.79 | 6286.81 | 7322.41 * |
| Ex4-1b | *v*, *a* (continuous *v*) | 5464.60 | 8270.34 | 5511.91 | 6415.61 ** |
| **Indicates the model that is reported in the manuscript.*  ***Indicates the best-performing model.* | | | | | |

*Posterior distributions of DDM parameters.* We determined the statistical significance of DDM parameters using a Bayesian estimation method, which is similar to a frequentist t-test (Kruschke, 2013). We calculated the probability that a randomly-sampled value from the target posterior distribution would fall above (or below) a specified cutoff (akin to a t-test against zero), or against the mean of another distribution. Mean differences and significant differences are reported in the main manuscript; the raw posterior distributions are reported here for the models treating the number of punishers as a categorical variable (Figure S6A-C) and as a continuous variable (Figure S6D-E).

***Figure S6 | DDM parameter posteriors when the number of punishers is categorical (top row) and continuous (bottom row).*** ***A)*** *Categorical bias. The vertical dashed line indicates the location of 0.5, which represents no bias for either compensation or punishment. Values less than 0.5 indicate a bias for compensation, and values greater than 0.5 indicate a bias for punishment.* ***B)*** *Categorical threshold. The “Alone” condition is used as the reference category, and all other posterior distributions represent regression coefficients. Negative values indicate a relatively lower threshold, and positive values indicate a relatively higher threshold.* ***C)*** *Categorical drift rate. As before, the “Alone” condition is used as the reference category. Negative values indicate relative evidence accumulation in favor of compensation, and positive values indicate relative evidence accumulation in favor of punishment. The magnitude of regression coefficients indicates the strength of evidence accumulation.* ***D)*** *Continuous bias.* ***E)*** *Continuous threshold. The proportion of punishers is defined as a categorical variable to capture the predicted nonlinear effect, and the condition with two punishers is used as the reference category. Units on the y-axis are arbitrary, as these are density plots.*

*Parameter recovery.* To ensure that HDDM is capable of accurately estimating the specific combination of parameters reported in our results given trial count limitations, we performed parameter recovery simulations using worst-case and best-case scenarios (20 and 100 trials per trial type, respectively). We generated data from 40 simulated subjects using the parameters estimated from our empirical data, assuming a subject noise scaling factor of 0.1 (HDDM’s default). We then performed parameter estimation on those simulated data using the exact same model that had previously been used to estimate parameters using empirical data. Details of the model fitting process can be found in the Supplementary Methods below. In summary, the known parameter means were contained in the simulated models’ 95% credible interval for each parameter of interest (14 per model, 42 per study, 84 in total). The two exceptions were: a) Victims’ *v* for Mildly Unfair offers and 2 punishers in the worst-case scenario (known mean 0.08, HDI range ﻿0.09-﻿0.32), and b) Victims’ *v* for Highly Unfair offers and 2 punishers in the worst-case scenario (known mean ﻿-0.16, HDI range ﻿-0.16-0.03, overlap caused by rounding error). However, given that the simulated data do not unduly deviate from the empirical findings, this does not hinder interpreting the estimated model parameters. For all models, 95% credible intervals of the posterior distributions were calculated using the HDInterval package in R (Meredith & Kruschke, 2016).

*Posterior predictive check: reaction times.* To examine how closely our model predictions mapped onto our observed data, we performed posterior predictive checks (PPC). We performed 1,000 simulations. In each of these simulations, parameter values for drift rate, threshold, and bias were randomly sampled from the posteriors of our parameter estimations. Each simulation returned a predicted choice and reaction time for every trial that each subject completed. In other words, a single simulation returned a dataset that precisely mirrored the structure of our observed dataset. After repeating this process 1,000 times, all simulated datasets were combined to analyze RT density and choice percentages (Figures S7 and S9).

***Figure S7 | Posterior predictive check of reaction times.*** *For visualization purposes, we plot only the time window ranging from 0-3 seconds. Jaggedness in the “observed” data reflects that subjects contributed different amounts of data to the model due to RT clipping. A more representative visualization of behavioral RT distributions can be found in Figure S5.*

The PPC reveals one particularly noteworthy pattern: The Victim model’s predictions least faithfully capture the observed RT distribution for Reverse decisions in the condition where offers are Mildly Unfair and the group unanimously prefers Compensation (i.e., 0% endorsement of punishment). We believe that the mismatch between the model prediction and observed data is an artifact of the Bayesian hierarchical fitting process. That is, if a minority of subjects were using a different decision strategy from the rest of the group, the hierarchical model would down-weight those subjects’ contributions to the group posterior. Therefore, if the majority of Reverse responses in specific conditions came from that minority group, we would expect to observe a discrepancy in RT distributions between the observed data (primarily composed of responses from the minority group) and the simulated data (relying more heavily on the group prior after discounting the contributions of the minority group).

To examine whether this might be the case, we plotted each subject’s proportion of Reverse responses in each cell (Figure S8A). We found that there was indeed a minority of subjects who endorsed punishment in the condition of interest (Mildly Unfair offers with no punishers). To see whether these particular subjects (four subjects in total) may have been using a different decision strategy from other subjects, we looked at qualitative data from our debriefing protocol. Indeed, we found that these subjects indicated a decision strategy that was different from the rest of our subjects: they all stated that they reversed more when offers were more fair, and compensated more when offers were more unfair (e.g., “Compensated when there was a big difference [i.e. when offers were more unfair] and Reversed when the proportions were closer together [i.e. when offers were more fair].”)

We consider this convincing evidence that the discrepancies revealed by the PPC are attributable to the hierarchical nature of our fitting process down-weighting the contributions of the minority of subjects who used a different decision strategy from other subjects. Because these subjects were responsible for the vast majority of Reverse choices in this condition, we do not attempt to make strong inferences about the cognitive processes underlying decisions to punish in this condition. For Jurors, we again find that there is a minority of subjects who endorse punishment in this condition (Figure S8B), which we again interpret as an artifact of the Bayesian hierarchical estimation process imposing its priors on the fitted parameters.

***Figure S8 |Each subject’s punishment rates by condition.*** *The x-axis lists each subject in our analysis. The y-axis represents the rate of punitive decisions. The dashed line represents the point at which a subject would be indifferent between punishment and compensation, which is 50% in our task.*

*Posterior predictive check: punishment rates.* In addition to simulating RTs, the PPC also simulated punishment rates (Figure S9). For Victims, we observe that our model overestimates punishment rates when there is a majority of compensators, and underestimates punishment rates when there is a majority of punishers. These subtle deviances suggest that our model may not adequately describe the range of punishment rates observed in the experiment. Formal tests reveal an interaction between data type (empirical versus simulated data) and the proportion of punishers for Victims (rmANOVA IV=punishment, DVs=proportion of punishers, offer unfairness, and data type, *F*(5, 195) = 4.66, Greenhouse-Geisser ϵ = ﻿0.32, *p* = 0.019, η^2^ = 0.005). However, we note that the deviance in the model’s predictions indicates an attenuated conformity effect, which biases the model results *against* our hypothesis. For Jurors, we observe that the data type (empirical versus simulated data) does not interact with the proportion of punishers (rmANOVA IV=punishment, DVs=proportion of punishers, offer unfairness, and data type, *F*(5, 210) = 2.50, Greenhouse-Geisser ϵ = ﻿0.26, *p* = 0.111, η^2^ = 0.002).

***Figure S9 | Posterior predictive check of punishment choices.*** *Error bars reflect ± 1 SEM.*

**SUPPLEMENTARY METHODS**

*Experiment 1: Last Decider*

Before beginning the Solo Phase, subjects were told that they would have the opportunity to make money by participating in economic interactions with past participants who had completed a similar study. After being assigned the role of Player B, subjects were further informed that they would be interacting with a different Player A on each trial and that they would never encounter the same Player A twice, in accordance with convention in behavioral economics to make all interactions anonymous and one-shot (Camerer, 2003). To ensure that subjects felt as if their decisions had real consequences for other people (FeldmanHall et al., 2012), they were informed that we had collected the mailing addresses of these past participants, and that we would be sending checks to past participants based on the choices that subjects made in the present session. Subjects were informed that the computer would determine payout by randomly selecting one trial from all the trials they completed. To prevent subjects from inferring any ulterior motives behind Player As’ offers, we additionally told subjects that the computer would pay out the selected trial according to Player A’s original offer half of the time, and according to Player B’s redistribution the other half. While the first 30 subjects received this instruction only in the Group Phase, all subjects in Experiment 3 received this instruction in both Solo and Group Phases. Critically, regardless of this oversight in the Solo Phase for the first 30 subjects, we observe a complete replication of the behavioral results in Experiment 3. Given that the task structure of Experiment 3 is closely related to the structure of Experiment 1, we take this as strong evidence that the difference in instruction did not have an effect on subjects’ strategies. To ensure that subjects understood all instructions, we administered a verbal quiz prior to beginning the task.

Throughout the task, subjects used the number keys 1-3 to respond, and choices were randomly re-mapped onto those keys on a trial-by-trial basis. Decisions were self-paced in the Solo Phase. In the Group Phase, subjects had up to eight seconds to make a choice, after which the computer automatically selected the Accept option and moved on to the next trial.

To increase the believability of the social deception during the Group Phase, we included photographs of these Player Bs in the task. Unbeknownst to subjects, these Player Bs were not real; Player B responses were computer-generated and photographs of fictitious partners were drawn from the Chicago Face Database and the MR2 database (Ma, Correll, & Wittenbrink, 2015; Strohminger et al., 2016). Due to past work demonstrating that people’s behaviors change in economic games when interacting with racial minorities and women (Kubota, Li, Bar-David, Banaji, & Phelps, 2013; Solnick, 2001), we only used photographs of white men. Subjects played with a different group on each trial. On average, subjects saw each Player B’s face in approximately six different groups. As we did in the instructions for the Solo Phase, we told subjects that we had collected the mailing addresses of all Player As and Player Bs they would encounter, and that we would send all players checks depending on the decisions that were made in the experimental session. Subjects were informed that their responses from that session would be forwarded to future participants, and to increase the believability of the social deception, we took a photograph of each participant before starting the Group Phase.

Due to an error in the paradigm, 25 subjects saw the same trial sequence, while the remaining 16 subjects each saw a fully-randomized trial sequence. Although we cannot definitively rule out the possibility that trial sequence may have a confounding effect on our results, we observe a full replication of our results in Experiment 3 (DDM), suggesting that the conformity effect we observe in Experiment 1 is robust to contextual factors such as trial sequence.

*Experiment 2: First Decider*

As in Experiment 1, to avoid subjects inferring ulterior motives underlying Player A offers, subjects were told that payouts were probabilistic such that Player A offers would be enacted 50% of the time, and that Player B redistribution would be enacted 50% of the time. However, subjects only received this instruction at the beginning of the Group Phase, and we cannot rule out the possibility that this may have had an impact on subjects’ punishment behaviors. Due to an error in the paradigm, nine subjects saw the same trial sequence, and the remaining 39 subjects each saw a fully-randomized trial sequence.

*Experiments 3-4: DDM*

As in Experiments 1 and 2, subjects in the Experiment 3 completed a Solo Phase followed by a Group Phase. To ensure that our Group Phase DDM results were not confounded by factors unrelated to the decision-making process (such as motor learning or learning a model of the task structure), subjects first completed 45 Solo Phase trials. We treat these Solo Phase trials as training trials: we did not analyze these data, and we do not report the results. Instead of using the training trials as our reference category, we interspersed 45 “Alone” trials during the Group Phase of our task. This “Alone” trial type is used as the reference category for all reported DDM regressions, and is also used as the baseline condition for all reported behavioral data (just as the “Solo Phase” trials were used as a baseline in Experiments 1 and 2).

Subjects completed six practice trials before beginning the Group Phase to ensure that DDM parameters would not be contaminated by unrelated task learning. To further guard against DDM parameter contamination due to start-up or task-switching costs, we added three “burn-in” trials after every break that subjects took. All burn-in trials are excluded from our analysis, and they do not count towards the final count of 420 trials in the Group Phase. After completing 1/3rd and 2/3rds of Group Phase trials, subjects were permitted to take self-timed breaks to combat potential effects of fatigue. Subjects were cued about the kind of trial they would complete (i.e. alone or with a group) by text reading either “ALONE” or “GROUP” for 0.5 seconds preceding each trial screen.

­

In the Group Phase of the task, subjects were simultaneously presented with Player A’s offer and what they believed were four randomly-sampled responses from past Player Bs (although as in Experiments 1 and 2, all Player A and Player B responses were computer-generated). Once again, subjects were told that Player B’s collective response would be determined by a simple majority of the five Player Bs on each trial. Finally, to reduce any potential task demands related to face processing and social learning, past Player Bs were simply represented as dots inside of boxes labeled Compensate and Reverse; subjects were told that the number of dots within the Compensate box and the Reverse box indicated the number of past participants who had espoused that option in each random sample of four past Player Bs.

The response key mapping was counterbalanced across participants and remained consistent across trials and across task phases. Subjects had an unlimited amount of time to decide, but were encouraged to respond as quickly as possible. Once a subject made a response, they were shown a feedback screen showing the outcome of their choice if Player B’s decision were enacted, though subjects were aware that the final payout was probabilistic such that Player A’s original offer would be enacted half of the time, and Player B’s collective decision would be enacted the other half of the time.

We performed hierarchical Bayesian parameter estimation using HDDM, a free and open-source software package (Wiecki et al., 2013). HDDM uses priors derived from past empirical literature to inform DDM parameters, and uses a Markov Chain Monte Carlo (MCMC) sampling method to estimate the joint posterior distributions of all parameters given the likelihood of observing empirical choices and RTs under a user-specified model. Technical details can be found in the HDDM methods paper. We obtained 10,000 samples of each posterior distribution to obtain smooth parameter estimates. For each model reported in our results, we generated a total of 10,000 samples of each posterior distribution using five separate MCMCs, each with an overall chain length of 2,200 iterations (the first 200 iterations were discarded as part of the “burn-in” process). We assessed MCMC convergence using the Gelman-Rubin scale reduction factor, which compares the variability within individual MCMCs and the variability between multiple independent MCMCs (Gelman & Rubin, 1992). As is conventional for Bayesian estimation, we ensured that all reported models had scale reduction factors of less than 1.1 for all parameters.

*Experiment 5: Crime ratings*

Subjects were assigned to read vignettes in which they were either the Victim or Juror. The trial sequence was pre-randomized. To control for potential gender effects, Jurors were asked for their gender at the beginning of the study. Men were shown vignettes in which men were always the victims, and women were shown vignettes in which women were always the victims. For each conjunction of crime type (assault versus theft), severity (low versus low), and group endorsement of punishment (0% to 100% endorsement), subjects saw two vignettes. They were written so that the gender of the perpetrator was balanced (i.e., the perpetrator was a man in one vignette and a woman in the other vignette). The names of victims were drawn from the United States Social Security Administration’s list of most popular names to avoid salience effects. Perpetrators were always unnamed. A complete list of the vignettes used can be found in Table S4.

***Table S4 | Crime vignettes.***

| **Crime Type** | **Victim** | **Female Juror** | **Male Juror** |
| --- | --- | --- | --- |
| Low-Intensity Assault | Your neighbor was hosting a loud party late at night. When you asked them to turn down the music, they slapped you in the face. | Susan's neighbor was hosting a loud party late at night. When Susan asked him to turn down the music, he slapped Susan in the face. | Robert's neighbor was hosting a loud party late at night. When Robert asked him to turn down the music, he slapped Robert in the face. |
|  | A drunken person stumbled outside of a bar, shoved you to the ground, and then rushed away unsteadily. | A drunken woman stumbled outside of a bar, shoved Barbara to the ground, and then rushed away unsteadily. | A drunken woman stumbled outside of a bar, shoved William to the ground, and then rushed away unsteadily. |
|  | During a basketball game, you got into an argument with a fan of the other team. They shoved you hard enough to make you fall. | During a basketball game, Nancy got into an argument with a fan of the other team. She pushed Nancy hard enough to make Nancy fall. | During a basketball game, Thomas got into an argument with a fan of the other team. She pushed Thomas hard enough to make Thomas fall. |
|  | You and a work colleague got into a heated argument about a project. They pushed you backwards into a wall. | Jennifer and a work colleague got into a heated argument about a project. He pushed Jennifer backwards into a wall. | Chris and a work colleague got into a heated argument about a project. He pushed Chris backwards into a wall. |
|  | A pedestrian ran into you on the sidewalk. After getting into an argument, they tripped you, causing you to fall to the ground. | A pedestrian ran into Margaret on the sidewalk. After getting into an argument, she tripped Margaret, causing Margaret to fall to the ground. | A pedestrian ran into Richard on the sidewalk. After getting into an argument, she tripped Richard, causing Richard to fall to the ground. |
|  | You got into an argument with a construction worker who obstructed your path. They kicked your leg when you tried to walk around. | Jessica got into an argument with a construction worker who obstructed Jessica's path. She kicked Jessica's leg when Jessica tried to walk around. | Michael got into an argument with a construction worker who obstructed Michael's path. She kicked Michael's leg when Michael tried to walk around. |
|  | During a dispute between you and your neighbor, they pushed you hard enough that you lost your balance. | During a dispute between Karen and her neighbor, he pushed Karen hard enough that Karen lost her balance. | During a dispute between John and his neighbor, he pushed John hard enough that John lost his balance. |
|  | You bumped into somebody while walking through a crowd. They got angry at you and punched you. | Elizabeth bumped into a man while walking through a crowd. He got angry at Elizabeth and punched Elizabeth. | Joseph bumped into a man while walking through a crowd. He got angry at Joseph and punched Joseph. |
|  | As you were boarding a bus, you unintentionally cut someone in line. They grabbed your arm and pulled you to the ground. | As Linda was boarding a bus, she unintentionally cut a woman in line. That woman grabbed Linda's arm and pulled her to the ground. | As James was boarding a bus, he unintentionally cut a woman in line. That woman grabbed James' arm and pulled him to the ground. |
|  | You overheard someone saying something mean about you. When you confronted them about it, they slapped you. | Mary overheard a man saying something mean about her. When Mary confronted him about it, he slapped Mary. | Charles overheard a man saying something mean about him. When Charles confronted him about it, he slapped Charles. |
|  | A coworker got angry at you because they thought you were interfering with their project. They hit you with an open fist. | Patricia's coworker got angry because she thought Patricia was interfering with her project. She hit Patricia with an open fist. | Daniel's coworker got angry because she thought Daniel was interfering with her project. She hit Daniel with an open fist. |
|  | While attending a live rock concert, you were hit in the face by a drunk person dancing wildly. | While attending a live rock concert, Sarah was hit in the face by a drunk man dancing wildly. | While attending a live rock concert, David was hit in the face by a drunk man dancing wildly. |
| High-Intensity Assault | During a football game, a heated argument erupted between you and another fan. In anger, they pulled out a pocketknife and stabbed you. | During a football game, a heated argument erupted between Emily and another fan. In anger, she pulled out a pocketknife and stabbed Emily. | During a football game, a heated argument erupted between Kevin and another fan. In anger, she pulled out a pocketknife and stabbed Kevin. |
|  | You confronted a coworker who was not contributing to a team project. They got angry and threw a stapler, which struck your head. | Carol confronted a coworker who was not contributing to a team project. He got angry and threw a stapler, which struck Carol's head. | Mark confronted a coworker who was not contributing to a team project. He got angry and threw a stapler, which struck Mark's head. |
|  | You had a dispute with a driver after they crashed into the back of your car. They took out a tire iron and beat you with it. | Melissa had a dispute with a driver after she crashed into the back of Melissa's car. She took out a tire iron and beat Melissa with it. | Andrew had a dispute with a driver after she crashed into the back of Andrew's car. She took out a tire iron and beat Andrew with it. |
|  | You and your restaurant server got into an argument. They hit you over the head with their serving tray so hard that it gave you a concussion. | Betty and her restaurant server got into an argument. She hit Betty over the head with the serving tray so hard that it gave Betty a concussion. | Paul and his restaurant server got into an argument. She hit Paul over the head with the serving tray so hard that it gave Paul a concussion. |
|  | You got into a fight with a stranger at the bar who had a lot to drink. They broke a bottle and stabbed you with the jagged glass. | Michelle got into a fight with a stranger at the bar who had a lot to drink. She broke a bottle and stabbed Michelle with the jagged glass. | Brian got into a fight with a stranger at the bar who had a lot to drink. She broke a bottle and stabbed Brian with the jagged glass. |
|  | During a community baseball game, you got into a violent quarrel with a teammate. They attacked you with a baseball bat. | During a community baseball game, Dorothy got into a violent quarrel with a teammate. He attacked Dorothy with a baseball bat. | During a community baseball game, Donald got into a violent quarrel with a teammate. He attacked Donald with a baseball bat. |
|  | You got into a political argument with your colleague while at dinner. They were so angry that they grabbed a steak knife and stabbed you. | Donna got into a political argument with a colleague while at dinner. He was so angry that he grabbed a steak knife and stabbed Donna. | George got into a political argument with a colleague while at dinner. He was so angry that he grabbed a steak knife and stabbed George. |
|  | After accidentally bumping into someone at the gym, you got into a fight. They picked up a heavy dumbbell and hit you with it. | After accidentally bumping into a woman at the gym, Sandra got into a fight. The woman picked up a heavy dumbbell and hit Sandra with it. | After accidentally bumping into a woman at the gym, Matthew got into a fight. The woman picked up a heavy dumbbell and hit Matthew with it. |
|  | You got into a fight with someone in your office. They grabbed a pair of scissors from the table and stabbed you with them. | Lisa got into a fight with a man in her office. He grabbed a pair of scissors from the table and stabbed Lisa with them. | Anthony got into a fight with a man in his office. He grabbed a pair of scissors from the table and stabbed Anthony with them. |
|  | During an argument with a hardware store employee, they picked up a shovel and hit you in the stomach with it. | When Amanda got into an argument with a hardware store employee, he picked up a shovel and hit Amanda with it. | When Kenneth got into an argument with a hardware store employee, he picked up a shovel and hit Kenneth with it. |
|  | You drunkenly spilled a drink onto someone at the bar. They got angry and cut your hands with a knife as you defended yourself. | Ashley drunkenly spilled a drink onto a man at the bar. The man got angry and cut Ashley's hands with a knife as Ashley defended herself. | Steven drunkenly spilled a drink onto a man at the bar. The man got angry and cut Steven's hands with a knife as Steven defended himself. |
|  | In a dispute with a neighbor that escalated into a fight, they pulled out a gun and repeatedly hit you in the head with it. | In a dispute between neighbors that escalated into a fight, a woman pulled out a gun and repeatedly hit Kimberly in the head with it. | In a dispute between neighbors that escalated into a fight, a woman pulled out a gun and repeatedly hit Joshua in the head with it. |
| Low-Intensity Theft | During a bag snatching, a thief ran off with your backpack, which contained your laptop. | During a bag snatching, a man ran off with Helen's backpack, which contained Helen's laptop. | During a bag snatching, a man ran off with Timothy's backpack, which contained Timothy's laptop. |
|  | You were listening to music while taking a walk when someone grabbed your phone and took off in the other direction. | Stephanie was listening to music while taking a walk when a woman grabbed Stephanie's phone and took off in the other direction. | Jacob was listening to music while taking a walk when a woman grabbed Jacob's phone and took off in the other direction. |
|  | While you were waiting in line to check out at the supermarket, a pickpocket lifted your wallet from your coat pocket. | While Sharon was waiting in line to check out at the supermarket, a woman lifted Sharon's wallet from Sharon's coat pocket. | While Edward was waiting in line to check out at the supermarket, a woman lifted Edward's wallet from Edward's coat pocket. |
|  | When stopped at a red light, a thief opened your car door and took your laptop from the backseat before making a getaway. | When stopped at a red light, a woman opened Laura's car door and took Laura's laptop from the backseat before making a getaway. | When stopped at a red light, a woman opened Nick's car door and took Nick's laptop from the backseat before making a getaway. |
|  | As you were sleeping in the middle of a flight, a fellow passenger stole your wallet and your cell phone. | As Amy was sleeping in the middle of a flight, a man stole Amy's wallet and cell phone. | As Gary was sleeping in the middle of a flight, a man stole Gary's wallet and cell phone. |
|  | You were walking through the park when a stranger jumped you, taking your wallet and phone before fleeing. | Kathleen was walking through the park when a woman jumped her, taking Kathleen's wallet and phone before fleeing. | Stephen was walking through the park when a woman jumped him, taking Stephen's wallet and phone before fleeing. |
|  | While you were waiting for the pedestrian walk signal, a passing bicyclist grabbed your bag from your hands before riding off. | While Anna was waiting for the pedestrian walk signal, a woman biking past grabbed Anna's bag from her hands before riding off. | While Jason was waiting for the pedestrian walk signal, a woman biking past grabbed Jason's bag from his hands before riding off. |
|  | On your way to deposit cash into an ATM, a thief waiting nearby jumped you and took all of your cash. They then fled the scene. | On Angela's way to deposit cash into an ATM, a man waiting nearby jumped Angela and took all of Angela's cash. He then fled the scene. | On Eric's way to deposit cash into an ATM, a man waiting nearby jumped Eric and took all of Eric's cash. He then fled the scene. |
|  | While you were using your phone at the subway station, someone snatched it from your hands before slipping away into the crowd. | While Cynthia was using her phone at the subway station, a man snatched it from Cynthia's hands before slipping away into the crowd. | While Jeff was using his phone at the subway station, a man snatched it from Jeff's hands before slipping away into the crowd. |
|  | In a crowded amusement park, someone bumped into you so hard you nearly fell. Later that day, you found out that they stole your wallet. | In a crowded amusement park, a man bumped into Deborah so hard she nearly fell. Later that day, Deborah found out that he stole her wallet. | In a crowded amusement park, a man bumped into Ryan so hard he nearly fell. Later that day, Ryan found out that he stole his wallet. |
|  | While working in a coffeeshop, you left your laptop to use the bathroom. When you got back, you found someone stealing it in your absence. | While working in a coffeeshop, Shirley left her laptop to use the bathroom. When she got back, she saw a woman stealing it in her absence. | While working in a coffeeshop, Jonathan left his laptop to use the bathroom. When he got back, he saw a woman stealing it in his absence. |
|  | Someone asked if they could borrow your phone to make a call. After you handed them your phone, they rushed off with it. | A man asked Rebecca if he could borrow Rebecca's phone to make a call. After Rebecca handed him the phone, he rushed off with it. | A man asked Ron if he could borrow Ron's phone to make a call. After Ron handed him the phone, he rushed off with it. |
| High-Intensity Theft | You were walking alone when someone armed with a hunting knife stole your bag. They ran away after taking it. | Catherine was walking alone when a woman armed with a hunting knife stole her bag. The woman ran away after taking it. | Brandon was walking alone when a woman armed with a hunting knife stole his bag. The woman ran away after taking it. |
|  | Someone put a gun to your chest and demanded that you give them your cash. After taking your money, they fled. | A man put a gun to Christine's chest and demanded that Christine give up her cash. After taking Christine's money, the man fled. | A man put a gun to Raymond's chest and demanded that Raymond give up his cash. After taking Raymond's money, the man fled. |
|  | A thief armed with a knife grabbed your bag from your shoulder before escaping into the crowd. Your bag had contained your laptop. | A woman armed with a knife grabbed Janet's bag from her shoulder before escaping into the crowd. Janet's bag had contained Janet's laptop. | A woman armed with a knife grabbed Scott's bag from his shoulder before escaping into the crowd. Scott's bag had contained Scott's laptop. |
|  | While you were walking to the coffeeshop, a person stole your phone at knifepoint, then bolted in the opposite direction. | While Brenda was walking to the coffeeshop, a woman stole Brenda's phone at knifepoint, then bolted in the opposite direction. | While Larry was walking to the coffeeshop, a woman stole Larry's phone at knifepoint, then bolted in the opposite direction. |
|  | While you were in a convenience store, a robber came and took your cash at gunpoint. They got into a getaway car and drove away. | While Katherine was in a convenience store, a man came and took Katherine's cash at gunpoint. He got into a getaway car and drove away. | While Justin was in a convenience store, a man came and took Justin's cash at gunpoint. He got into a getaway car and drove away. |
|  | Someone pressed a gun into your back and told you to give up your wallet and watch. You heard them fleeing afterwards. | A man pressed a gun into Virginia's back and told her to give up her wallet and watch. Virginia heard the man fleeing afterwards. | A man pressed a gun into Alexander's back and told him to give up his wallet and watch. Alexander heard the man fleeing afterwards. |
|  | A person with a knife threatened to stab you unless you gave them your phone. They escaped with it before you could call for help. | A woman with a knife threatened to stab Samantha unless she gave up her phone. The woman escaped with it before Samantha could call for help. | A woman with a knife threatened to stab Jack unless he gave up his phone. The woman escaped with it before Jack could call for help. |
|  | While you were on vacation, an armed thief threatened to shoot you unless you gave them your camera. They ran away after they took it. | While Nicole was on vacation, an armed woman threatened to shoot Nicole unless she gave up her camera. The woman ran away after she took it. | While Gregory was on vacation, an armed woman threatened to shoot Gregory unless he gave up his camera. The woman ran away after she took it. |
|  | You pulled over to help a hitchhiker on a busy street. They pulled out a knife, forced you to give up your cash, and then took off. | Pam pulled over to help a hitchhiker on a busy street. He pulled out a knife, forced Pam to give up her cash, and then took off. | Ben pulled over to help a hitchhiker on a busy street. He pulled out a knife, forced Ben to give up his cash, and then took off. |
|  | After making a cash withdrawal from your bank, you were jumped by someone who robbed you at gunpoint before running away. | After Debra made a cash withdrawal from her bank, she was jumped by a woman who robbed Debra at gunpoint before running away. | After Patrick made a cash withdrawal from his bank, he was jumped by a woman who robbed Patrick at gunpoint before running away. |
|  | In the parking lot after work, you encountered a person with a knife. They took your work laptop, then made a getaway. | In the parking lot after work, Ruth encountered a man with a knife. He took Ruth's work laptop, then made a getaway. | In the parking lot after work, Samuel encountered a man with a knife. He took Samuel's work laptop, then made a getaway. |
|  | While you were using an ATM, an individual with a gun demanded that you give them your cash. They rushed off after stealing it. | While Rachel was using an ATM, a man with a gun demanded that Rachel give up her cash. The man rushed off after stealing it. | While Frank was using an ATM, a man with a gun demanded that Frank give up his cash. The man rushed off after stealing it. |

**SUPPLEMENTARY REFERENCES**

Barr, D. J., Levy, R., Scheepers, C., & Tily, H. J. (2013). Random effects structure for confirmatory hypothesis testing: Keep it maximal. *Journal of Memory and Language, 68*(3), 255-278. doi:https://doi.org/10.1016/j.jml.2012.11.001

Bates, D., Mächler, M., Bolker, B., & Walker, S. (2015). Fitting Linear Mixed-Effects Models Using lme4. *Journal of Statistical Software; Vol 1, Issue 1 (2015)*.

Camerer, C. (2003). *Behavioral game theory: Experiments in strategic interaction*. Princeton, NJ: Princeton University Press.

FeldmanHall, O., Mobbs, D., Evans, D., Hiscox, L., Navrady, L., & Dalgleish, T. (2012). What we say and what we do: The relationship between real and hypothetical moral choices. *Cognition, 123*(3), 434-441. doi:https://doi.org/10.1016/j.cognition.2012.02.001

FeldmanHall, O., Otto, A. R., & Phelps, E. A. (2018). Learning another's preference to punish enhances one's own punitive behavior. *Journal of Experimental Psychology: General*.

Gelman, A., & Rubin, D. B. (1992). Inference from Iterative Simulation Using Multiple Sequences. *Statistical Science, 7*(4), 457-472.

Kruschke, J. K. (2013). Bayesian estimation supersedes the t test (Vol. 142, pp. 573-603). US: American Psychological Association.

Kubota, J. T., Li, J., Bar-David, E., Banaji, M. R., & Phelps, E. A. (2013). The Price of Racial Bias: Intergroup Negotiations in the Ultimatum Game. *Psychological Science, 24*(12), 2498-2504. doi:10.1177/0956797613496435

Kuznetsova, A., Brockhoff, P. B., & Christensen, R. H. B. (2017). lmerTest Package: Tests in Linear Mixed Effects Models. *Journal of Statistical Software; Vol 1, Issue 13 (2017)*.

Ma, D. S., Correll, J., & Wittenbrink, B. (2015). The Chicago face database: A free stimulus set of faces and norming data. *Behavior Research Methods, 47*(4), 1122-1135. doi:10.3758/s13428-014-0532-5

Meredith, M., & Kruschke, J. K. (2016). HDInterval: Highest (Posterior) Density Intervals. Retrieved from https://CRAN.R-project.org/package=HDInterval

Sheather, S. (2009). *A modern approach to regression with R*: Springer Science & Business Media.

Solnick, S. J. (2001). Gender differences in the ultimatum game. *Economic Inquiry, 39*(2), 189-200. doi:10.1111/j.1465-7295.2001.tb00060.x

Strohminger, N., Gray, K., Chituc, V., Heffner, J., Schein, C., & Heagins, T. B. (2016). The MR2: A multi-racial, mega-resolution database of facial stimuli. *Behavior Research Methods, 48*(3), 1197-1204. doi:10.3758/s13428-015-0641-9

Wiecki, T., Sofer, I., & Frank, M. (2013). HDDM: Hierarchical Bayesian estimation of the Drift-Diffusion Model in Python. *Frontiers in Neuroinformatics, 7*, 14.
